# Activation of Innate and Adaptive Immunity by a Recombinant Human Cytomegalovirus Strain Expressing an NKG2D Ligand

**DOI:** 10.1371/journal.ppat.1006015

**Published:** 2016-12-01

**Authors:** Adriana Tomić, Pavankumar R. Varanasi, Mijo Golemac, Suzana Malić, Peggy Riese, Eva M. Borst, Eva Mischak-Weissinger, Carlos A. Guzmán, Astrid Krmpotić, Stipan Jonjić, Martin Messerle

**Affiliations:** 1 Institute of Virology, Hannover Medical School, Hannover, Germany; 2 Clinics of Hematology, Hemostasis, Oncology and Stem Cell Transplantation, Hannover Medical School, Hannover, Germany; 3 Department of Histology and Embryology, Faculty of Medicine, University of Rijeka, Rijeka, Croatia; 4 Department of Vaccinology and Applied Microbiology, Helmholtz Centre for Infection Research, Braunschweig, Germany; 5 German Center for Infection Research (DZIF), Partner Site Hannover-Braunschweig, Germany; Emory Vaccine Center, UNITED STATES

## Abstract

Development of an effective vaccine against human cytomegalovirus (HCMV) is a need of utmost medical importance. Generally, it is believed that a live attenuated vaccine would best provide protective immunity against this tenacious pathogen. Here, we propose a strategy for an HCMV vaccine that aims at the simultaneous activation of innate and adaptive immune responses. An HCMV strain expressing the host ligand ULBP2 for the NKG2D receptor was found to be susceptible to control by natural killer (NK) cells, and preserved the ability to stimulate HCMV-specific T cells. Infection with the ULBP2-expressing HCMV strain caused diminished cell surface levels of MHC class I molecules. While expression of the NKG2D ligand increased the cytolytic activity of NK cells, NKG2D engagement in CD8+ T cells provided co-stimulation and compensated for lower MHC class I expression. Altogether, our data indicate that triggering of both arms of the immune system is a promising approach applicable to the generation of a live attenuated HCMV vaccine.

## Author Summary

Human cytomegalovirus (CMV) is a major cause of morbidity and mortality in congenitally infected newborns and immunocompromised individuals, indicating an utmost need for a vaccine to protect these vulnerable groups. Recent experimental studies in animal models, including non-human primates, have shown that attenuated CMVs trigger a potent immune response and are attractive vaccine candidates. However, an effective CMV vaccine is still not available. Here, we demonstrate that rational engineering of a live attenuated human CMV vaccine candidate is feasible. We equipped a CMV strain with an immunostimulatory molecule that is a ligand for an activating receptor present on both Natural Killer cells and CD8+ T cells. Moreover, we deleted several immunoevasins involved in downregulation of MHC class I molecules and of a ligand for Natural Killer cells in order to elicit stronger immune responses. In vitro assays using human immune cells and a first assessment in a humanized mouse model in vivo suggest that the generated CMV strain is attenuated and has the ability to induce a virus-specific immune response. Our study proposes this novel approach for the development of a rationally engineered CMV vaccine.

## Introduction

Human cytomegalovirus (HCMV) is a highly prevalent herpesvirus infecting most of the human population [1, 2]. In immunocompetent individuals infection is usually asymptomatic, yet results in the establishment of latency. However, HCMV is the leading cause of congenitally related diseases causing severe and often irreparable birth defects [3–5]. HCMV is also the most common opportunistic infection causing morbidity in immunocompromised patients [6–8]. Due to the high economic and health burden, development of an HCMV vaccine has key public health priority [9, 10].

Vaccination of adolescents, or ideally of all children at young age, would be the most effective strategy to reduce the incidence of congenital CMV infection [11]. The immune correlates preventing transmission of CMV across the placenta are not completely defined yet; however, it seems likely that both humoral and cellular immunity are contributing to protection. Vaccination of immunocompromised transplant recipients would be more challenging, because of lower ability to mount immune responses and also due to safety concerns; nonetheless, at least in solid organ transplant patients, inducing or boosting immunity before transplantation would be feasible and promising. However, in both settings, clinical trials with subunit vaccines were only partially efficacious in preventing infection [12, 13]. Humoral and cellular immunity can more effectively be achieved by application of a live vaccine. Indeed several preclinical studies in animal models, including in non-human primates, revealed a robust capacity of attenuated CMVs to elicit a potent memory T cell response [14–20].

With the exception of the well-established Oka vaccine that provides excellent protection against varicella-zoster virus, no attenuated vaccine against other herpesviruses has been approved. There are several difficulties that hamper the development of an effective live HCMV vaccine. The ability of HCMV to reactivate and to re-infect seropositive individuals indicates that immunity resulting from primary infection cannot completely prevent subsequent infections [21–25]. Another challenge is to accomplish an adequate balance between safety and immunogenicity. For instance, a live HCMV vaccine based on the Towne strain could not prevent infection of renal transplant recipients, but lowered severity of CMV disease [26, 27], suggesting that this vaccine strain was over-attenuated. Thus, there is a need to rationally engineer an HCMV vaccine that induces comparable or ideally better immunity than natural infection and at the same time presents an excellent safety profile. One approach is the generation of chimeras between the Towne and Toledo strains and these are currently investigated in clinical trials [28, 29].

Numerous viral encoded immunoevasins prevent the development of full-blown CMV-specific immunity. Accordingly, genetically modified animal CMVs lacking immunoevasins exhibited outstanding vaccine properties in mouse and guinea pig models [30, 31]. Recently, we found that a mouse CMV (MCMV) expressing a host ligand for the activating receptor NKG2D induces an efficient CD8+ T cell response despite being profoundly attenuated [15, 32]. Moreover, challenge infection experiments indicated that the protection obtained after immunization is superior to the one seen after natural infection [15, 32].

It is highly desirable to test the suitability of similar approaches for a recombinant HCMV vaccine. However, the strategy successfully established for MCMV cannot simply be translated to HCMV. Divergence between rodents and primates started ~90 million years ago and resulted in some differences of the immune system of mouse and man [33]. Similarly, co-evolution with the respective hosts led to even larger dissimilarity of viral virulence factors [34, 35].

In this study, we report on the construction and evaluation of a recombinant HCMV strain expressing the host ligand ULBP2 for the NKG2D receptor. We found that cells infected with the ULBP2-expressing strain increased the cytolytic activity of NK cells in an NKG2D-dependent manner, thereby preventing viral spread and containing the viral infection to few cells. Still, the strain exhibited a robust capacity to activate HCMV-specific T cells. Interestingly, the mechanisms steering T cell activation turned out to be different for the viral strains. Altogether, our data imply that the chosen strategy allows attenuation of an HCMV strain, while retaining its ability to stimulate innate NK cell and adaptive T cell immunity.

## Results

### The ULBP2-expressing HCMV strain enhances NK cell-mediated cytotoxicity, limiting viral spread

**Fig 1:**
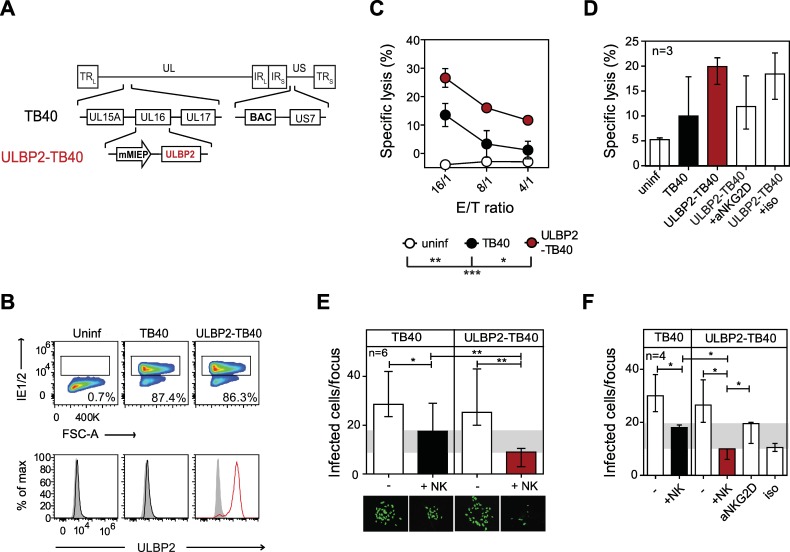
Spread of the ULBP2-expressing HCMV strain is strongly controlled by NK cells. (A) The ULBP2-TB40 strain was generated by insertion of a ULBP2 expression cassette (driven by the mouse CMV major immediate-early promoter (mMIEP)), replacing ORF UL16 in the BAC-cloned TB40 genome that lacks also the US1 to US6 region. (B) HFF were infected at an MOI of 1 with TB40 or ULBP2-TB40 or left uninfected. The infection rate at day 1 p.i. was determined by flow cytometry following intranuclear staining with an IE1/2-specific antibody. Lower panels, ULBP2 surface expression on uninfected, TB40 (black lines) or ULBP2-TB40 infected cells (red line). Grey fill, isotype control. (C) Cytotoxicity assays were set up at the indicated effector/target (E/T) ratios with primary NK cells and uninfected (white circles), TB40 (black circles) and ULBP2-TB40 (red circles) infected HFF (MOI 1, 1 d.p.i.). Results are means ± SEM of 3 independent experiments performed with NK cells of one donor. (D) Compiled data of cytotoxicity assays performed with NK cells from 3 HCMV-negative donors as in (C), and in addition with ULBP2-TB40 infected target cells in the presence of an NKG2D blocking antibody (aNKG2D) or isotype antibody (iso). Results are medians (with range) of specific lysis at E/T ratio 8:1 (n = 3 donors). Data were compiled from 3 independent experiments. (E) Focus expansion assays using TB40 or ULBP2-TB40 infected HFF and primary NK cells. At day 3 of co-culturing infected cells were detected by fluorescence microscopy following staining with an IE1/2 antibody. Graph displays cumulative data as medians (with range) of experiments done with NK cells of 6 donors. A focus of infection is defined as a cluster of 3 to 60 infected cells. Four wells were analyzed per donor and all infectious foci were counted with 2 to 8 foci being present per well. Images are from one representative experiment. (F) Focus expansion assays were carried out as described in (E) and additionally in the presence of NKG2D blocking (aNKG2D) or isotype antibodies (iso) using NK cells from 4 donors. Statistical analysis for (C) (NK cell-mediated cytotoxicity assay at E/T ratio 16:1) was done with two-way ANOVA with Bonferroni correction and for (E, F) with Mann-Whitney t-test. *, P < 0.05, **, P < 0.01, ***, P < 0.001.

To generate an HCMV strain for testing a novel vaccination approach that aims at activation of NK cells as well as T cells we used the bacterial artificial chromosome (BAC)-cloned strain TB40/E (herein referred as TB40) [36] that displays several features useful for vaccine development. In contrast to most other HCMV isolates, after extended passages in fibroblasts TB40 retains a complex of the glycoproteins gH/gL/UL128-UL131A (called pentameric complex) on the envelope of its virion important for broad cell tropism and produces a high rate of cell free virus, ultimately facilitating large scale production under GMP conditions [36]. TB40 lacks the immunoevasins US2, US3 and US6 [36] involved in MHC class I downregulation, which is expected to improve antigen presentation and priming of the CD8+ T cell response [37]. Note that US11 –another MHC class I immunoevasin–is retained. In order to attenuate the virus (named ULBP2-TB40) and to render it sensitive to NK cell control we inserted the gene for the host NKG2D ligand ULBP2 in the viral genome and replaced UL16, which normally interferes with expression of NKG2D ligands in infected cells [38–40] (Fig 1A). The other NK cell evasion genes present in the parental strain [36] were retained. Insertion of the ULBP2 open reading frame (ORF) had no influence on the viral growth kinetics in vitro (S1 Fig). In human foreskin fibroblasts (HFF) infected with ULBP2-TB40, strong ULBP2 surface expression was observed, whereas in cells infected with the parental strain TB40 no ULBP2 could be detected (Fig 1B). Expression of ULBP2 in HCMV infected cells led to substantially increased lysis by NK cells (for all donors tested) when compared to cells infected with TB40 (Fig 1C and 1D). Blocking of the NKG2D receptor with a specific antibody indicated that for NK cells of all donors analyzed the observed gain of cytotoxicity against ULBP2-TB40 infected cells was mediated via an NKG2D-dependent mechanism (Fig 1D). To determine the effect of the improved NK cell response on viral spread, we compared the ability of primary human NK cells to control the transmission of the viruses from infected to non-infected neighboring cells by applying a focus expansion assay [41]. NK cells could limit the spread of the TB40 virus as reported previously [41]. However, spread of the ULBP2 expressing strain was markedly more inhibited leading to less infected cells per focus of infection (Fig 1E) as well as a lower number of infectious foci in cell cultures (S2 Fig). Blocking of the NKG2D receptor with antibodies reduced the NK cell control of ULBP2-TB40 to the level observed for TB40 (Fig 1F), confirming that the observed additional gain of control is mediated by an NKG2D-dependent mechanism. Taken together, viral expression of ULBP2 leads to increased NK cell-mediated cytotoxicity that limits viral spread.

### A first in vivo assessment of the ULBP2-TB40 strain in humanized mice

**Fig 2:**
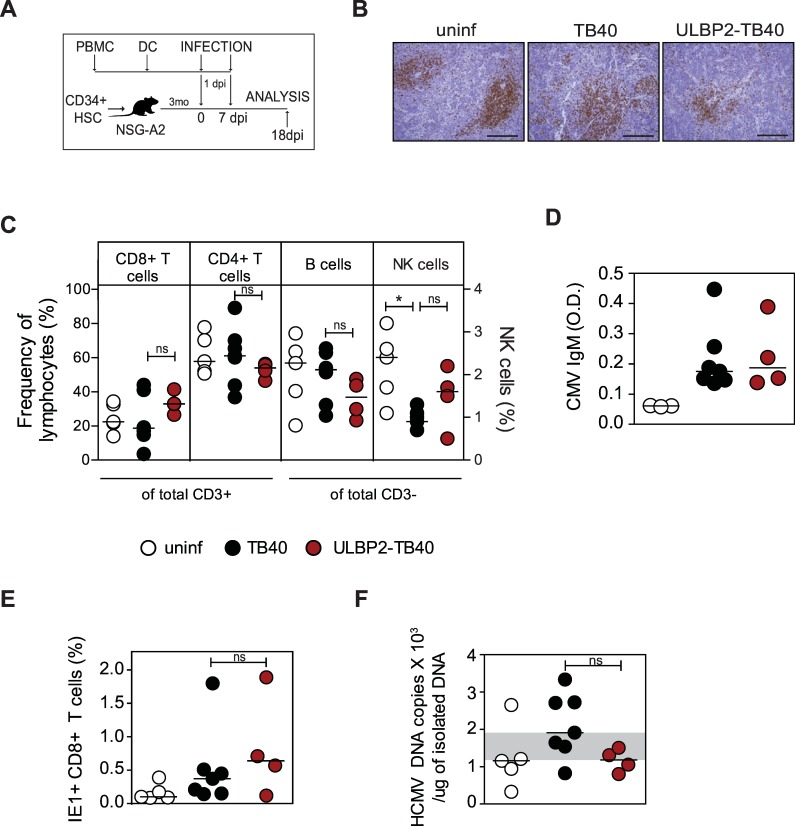
Immunogenicity of the ULBP2-TB40 strain in humanized mice. (A) Experimental design for immunization of humanized mice with the HCMV strains. DC generated from HLA-A2+ HCMV-negative donors were infected or left uninfected and 1 day p.i. injected into NSG-A2 humanized mice. Mice were boosted 7 days later in the same manner. On day 18 p.i. mice were sacrificed and the frequency of HCMV-specific CD8+ T cells and viral load were analyzed. (B) Representative immunohistological images of spleens from one animal of each group after staining with a CD3 antibody. Scale bar, 200 μm. (C) Percentages of human CD8+ and CD4+ T cells out of total CD3+ cells and of CD19+ B cells and CD56+ NK cells (right y-axis in red) out of total CD3- cells in spleens of animals from the uninfected group (white circles), or groups receiving TB40 (black circles) and ULBP2-TB40 infected DC (red circles). (D) CMV-specific IgM antibodies in serum of infected animals detected by ELISA. (E) Percentage of IE1-tetramer+ CD8+ T cells in spleens of animals for the indicated groups. (F) Viral load in spleen at day 18 p.i. Each circle represents the result for one animal and lines indicate medians. Data from all panels was analyzed using the Mann-Whitney t-test. *, P < 0.05. ns, P > 0.05.

Mice reconstituted with human hematopoietic stem cells can serve as models for infection with several human-specific viruses [42, 43] and the use of such a model has also been reported for HCMV [44]. Thus, to assess whether the ULBP2-TB40 strain can induce an immune response in vivo we performed infection experiments in humanized mice. For a pilot experiment NOD/SCID/IL2Rcγc mice expressing human HLA-A02:01 molecules and engrafted with fetal liver-derived human CD34+CD38- hematopoietic stem cells (humanized NSG-A2 mice) were injected with HCMV-infected fibroblasts as described [44]. Although viral DNA could be detected in spleens of infected animals, we did not find hints for the generation of HCMV-specific CD8+ T cells, probably due to only low numbers of human myeloid cells present in these mice [45]. Thus, for subsequent experiments we injected humanized mice with uninfected or TB40 or ULBP2-TB40 infected DC (Fig 2A). Human CD3+ T cells present in periarteriolar lymphoid sheaths (PALS) were found in spleens at day 18 p.i., when the humanized NSG-A2 mice were sacrificed and organs examined, irrespectively on whether mice were injected with uninfected or infected DC (Fig 2B). The frequency of human CD8+ and CD4+ T cells, as well as of CD19+ B cells and CD56+ NK cells in spleens was in a similar range for the groups receiving infected DC (Fig 2C). In livers slightly higher frequencies of NK cells were detected in the group receiving ULBP2-TB40 infected DC than in the group receiving TB40-infected DC (although the differences were not significant) (S3A Fig). Immunophenotyping of the NK cells in spleen revealed that mice injected with ULBP2-TB40 infected cells had a higher percentage of CD57+ NK cells when compared to mice receiving the parental virus TB40 (S3B Fig). Moreover, in some mice injected with ULBP2-TB40 DC IFNγ-expressing NK cells were found (S3C Fig). The CD57 marker is expressed on mature NK cells [46, 47] and the detection of IFNγ producing NK cells suggests that they were functional.

CMV-specific antibodies appear particularly important for prevention of congenital infection [48]. We therefore examined the ability of the ULBP2-TB40 strain to mount a humoral immune response. Although NSG humanized mice are not ideal for analyzing antibody responses [49, 50], CMV-specific IgM antibodies were detected at comparable levels in the serum of both groups following immunization with either strain (Fig 2D). We concluded that the ULBP2-TB40 strain elicits CMV-specific antibodies with similar efficiency as the TB40 strain.

Finally, we investigated whether CMV-specific T cells were primed in the humanized mice. When examined at day 14 p.i., CD8+ T cells specific for the HCMV epitopes NLVPMVATV and VLEETSVML (derived from the viral proteins pp65 and IE1) were found in blood of several animals (S4 Fig). Moreover, HCMV-specific CD8+ T cells were also detected in spleens at day 18 p.i. (Fig 2E and S5A Fig). The percentage of IE1-specific CD8+ T cells was comparable between both infected groups (Fig 2E), and this applied also to the absolute numbers of IE1-specific CD8+ T cells (S5A Fig). IFNγ positive CD8+ T cells could be detected at low level in few animals (S5B Fig) following stimulation with a specific peptide, confirming priming of IE1-specific CD8+ T cells. The majority of the CD8+ T cells displayed a phenotype of terminally differentiated effector memory (TEMRA) cells (S5C Fig), independently of the treatment applied.

To investigate to which extent the two strains can establish infection in the humanized animals, viral loads were measured in several organs on day 18 p.i. using quantitative PCR (qPCR). Viral DNA was found only in spleens of infected animals, and not in the other organs analyzed. In spleen of most of the animals infected with the TB40 virus viral DNA was detected (Fig 2F), whereas in the animals of the ULBP2-TB40 infected group the viral DNA loads were lower, although the difference in the viral loads was not statistically significant. Taken together, we concluded that the ULBP2-TB40 virus is able to prime HCMV-specific CD8+T cells and induce CMV-specific antibodies in humanized mice to a similar extent as the TB40 strain despite low viral loads.

### The ULBP2-TB40 strains retains the ability to stimulate virus-specific CD8+ T cells and other immune effector cells

**Fig 3:**
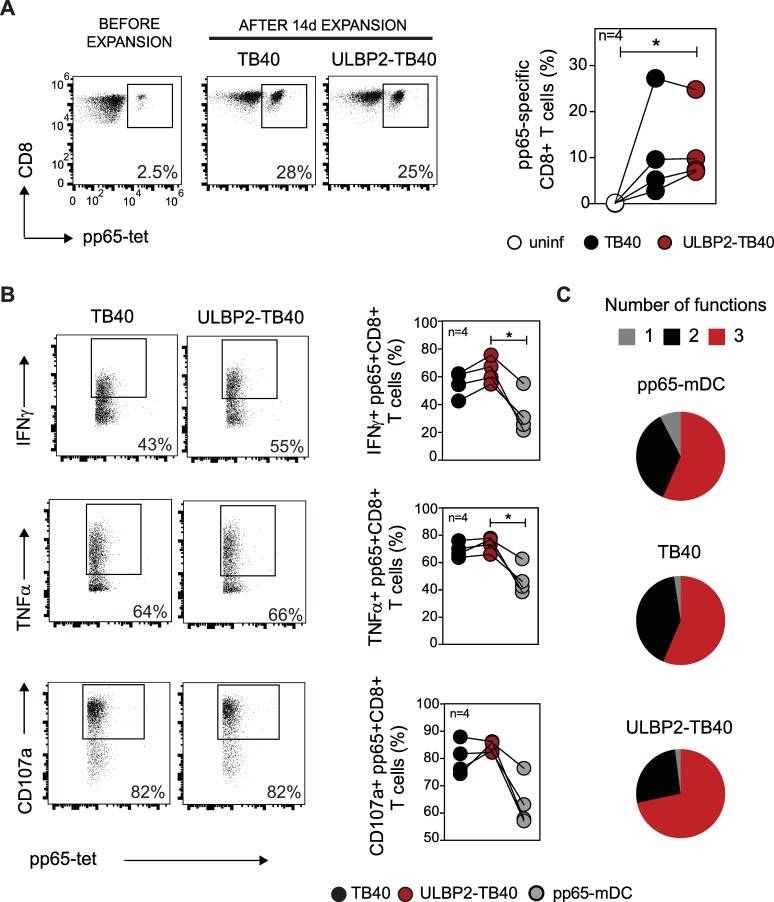
*Ex vivo* expansion with ULBP2-TB40 infected DC results in highly functional HCMV-specific CD8+ T cells. (A) Dot plots of an experiment performed with cells from one donor indicating percentage of pp65-specific CD8+ T cells before expansion and after 14 days of co-culture with TB40 or ULBP2-TB40 infected DC. Graph displays compiled data for four donors as percentages of pp65-specific CD8+ T cells expanded with uninfected (white circles), TB40 (black circles) and ULBP2-TB40 (red circles) infected DC. Data obtained with cells from individual donors are connected by lines. (B) Cytokine production and degranulation of pp65-specific CD8+ T cells (obtained as described in (A) or upon co-culture with pp65 peptide-loaded DC (pp65-mDC)) in response to pp65 peptide-loaded DC as target cells. Representative staining for IFNγ, TNFα and CD107a of pp65-specific CD8+ T cells obtained from one donor. The graphs at right are compiled data for 4 donors. (C) Multifunctionality of the expanded pp65-specific CD8+ T cells. Multifunctional subsets were determined using Boolean analysis based on the simultaneous measurement of IFNγ, TNFα and CD107a positive cells. Pie charts denote the proportion of pp65-specific CD8+ T cells having 1, 2 or 3 functions for 4 different donors. Data are representative of one out of two independent experiment performed. Statistical analysis was done with one-way ANOVA Friedman test followed by Dunn’s Multiple Comparison test. *, P < 0.05.

Encouraged by the observation that the ULBP2-TB40 strain could prime CD8 T cells in the humanized mice, we further investigated the activation of CMV-specific T cells by DC infected with this strain. It is well known that HCMV inhibits maturation of infected DC [51–53], and since DC are central for stimulation of T cells, it was important to know how the ULBP2-TB40 virus influences this process. In particular it was hard to predict whether expression of ULBP2 in HCMV infected DC increases the CD8+ T cell response by engaging the costimulatory NKG2D receptor [54, 55], or whether it would perhaps lead to T cell exhaustion [56]. To test the ability of TB40 and ULBP2-TB40 infected DC to expand HCMV-specific CD8+ T cells, primary CD8+ T cells isolated from HLA-A02:01 HCMV-seropositive healthy donors were co-cultured with infected autologous monocyte-derived DC or for control with uninfected DC (Fig 3). Mature DC loaded with the HLA-A02:01 specific immunodominant peptide NLVPMVATV derived from the pp65 protein were used as a positive control (S6 Fig). DC infected with either of the two viruses induced a comparable percentage of pp65-specific CD8+ T cells (Fig 3A). When tested against pp65 peptide-loaded DC, the pp65-specific CD8+ T cells had comparable cytokine production and degranulation capacity (Fig 3B). The simultaneous measurement of IFNγ, TNFα and degranulation allowed us to determine the multifunctionality of the pp65-specific CD8+ T cells, revealing that there was a trend toward higher multifunctionality of the T cells that were expanded with ULBP2-TB40 infected DC (Fig 3C), although the differences were not statistically significant. Expansion with ULBP2-TB40 infected DC resulted in a lower percentage of CD8+ T cells with central memory phenotype than expansion with pp65-peptide loaded DC (S7A Fig). Both viruses induced similar percentages of effector memory and central memory CD8+ T cells and of antigen-specific CD8+ T cells (S7A and S7B Fig). The frequency of exhausted PD-1+ CD8+ T cells after expansion with DC infected with either virus was in the same range (10 to 30% of total CD8+ T cells) (S7C and S7D Fig). Overall, we concluded that ULBP2-TB40-infected DC induced a comparable percentage and quality of HCMV-specific CD8+ T cells as TB40-infected DC.

**Fig 4:**
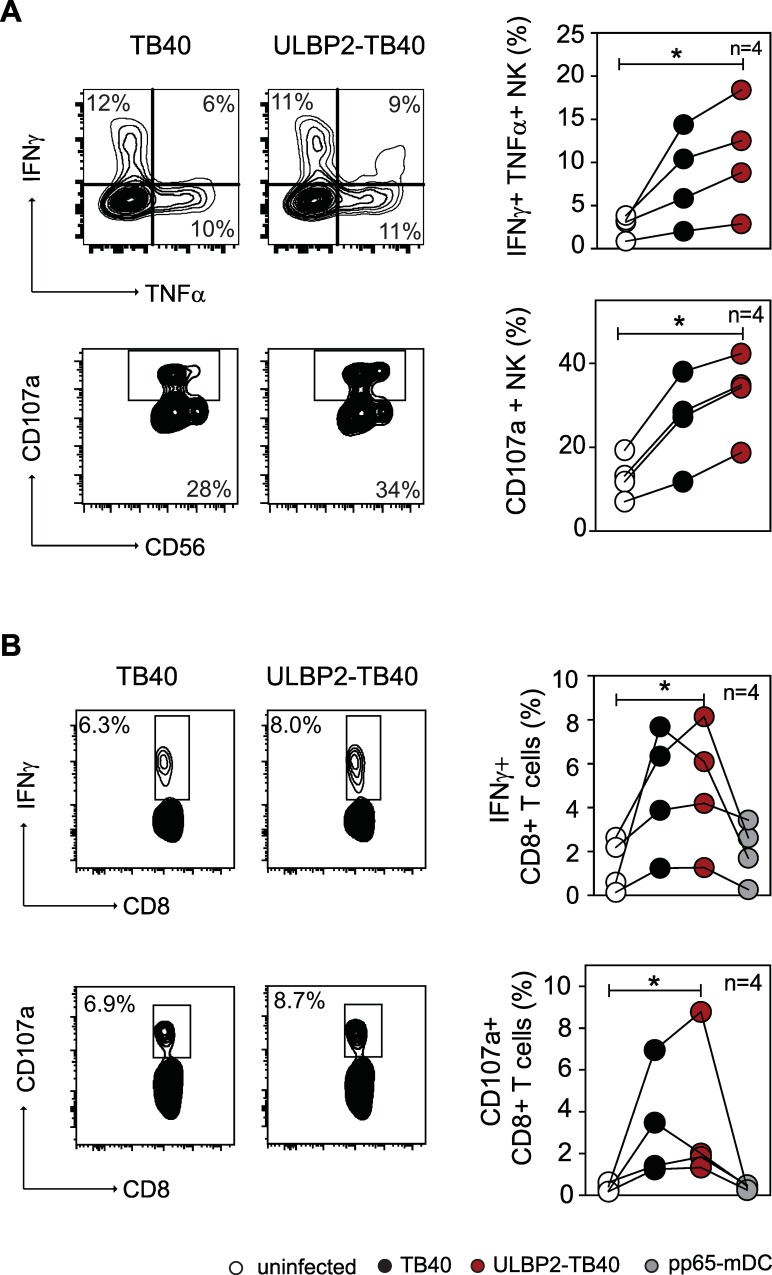
T cell activation with ULBP2-TB40 infected DC is not impaired in the presence of NK cells. Intracellular cytokine staining (IFNγ/TNFα or IFNγ) and degranulation (CD107a staining) of (A) NK cells (CD3-CD56+) and (B) CD8+ T cells (CD3+CD8+) upon co-cultivation of PBMC from one donor with autologous monocyte-derived DC treated or infected as indicated. Graphs on the right are compiled data of experiments performed with cells of 4 HCMV-seropositive donors. Data generated with cells of individual donors are connected by lines and are representative of one of three independent experiment performed. Statistical analysis was done with one-way ANOVA Friedman test followed by Dunn’s Multiple Comparison test. *, P < 0.05.

NK cells have the ability to modulate the outcome of T cell responses against CMV [57, 58]. They can influence activation of CMV-specific T cells in a positive manner by providing an appropriate microenvironment and by lysis of infected cells, thereby increasing the amount of available antigens [59, 60]. In contrast, NK cells can potentially also limit the activation of T cells by eliminating CMV infected DC [61, 62]. Since expression of the ULBP2 molecule by ULBP2-TB40 led to strong NK cell-mediated lysis of infected cells (compare Fig 1), it was important to analyze the effect of NK cells on the induction of T cell responses. To this end, we set up autologous stimulation assays using peripheral blood mononuclear cells (PBMC). PBMC isolated from HCMV-seropositive donors were co-cultured with autologous monocyte-derived DC either uninfected or infected with the respective viruses or–for control–matured and loaded with pp65 peptides. The infection rate of the DC was comparable for both viruses (S8A Fig). Activation of NK cells as assessed by intracellular cytokine staining for proinflammatory cytokines (IFNγ+, TNFα+) and degranulation (CD107a+) was in a similar range for cultures containing either ULBP2-TB40 or TB40-infected DC (Fig 4A). Likewise, the percentage of CD8+ T cells producing IFNγ+ and degranulating (CD107a+) was comparable upon co-cultivation with TB40 or ULBP2-TB40 infected DC (Fig 4B). Similar results were obtained for CD4+ T cells–ULBP2 expression did not impair the ability of CD4+ T cells to produce proinflammatory cytokines (IFNγ and TNFα) in response to infected DC (S8B and S8C Fig). In summary, PBMC co-cultures with ULBP2-TB40 infected DC activated CD8+ and CD4+ T cells as well as NK cells to the same extent as TB40-infected DC and most importantly, ULBP2 expression in the infected DC did not impair the stimulation of the immune effector cells.

### The ULBP2-TB40 strain causes stronger MHC class I downregulation in infected cells

**Fig 5:**
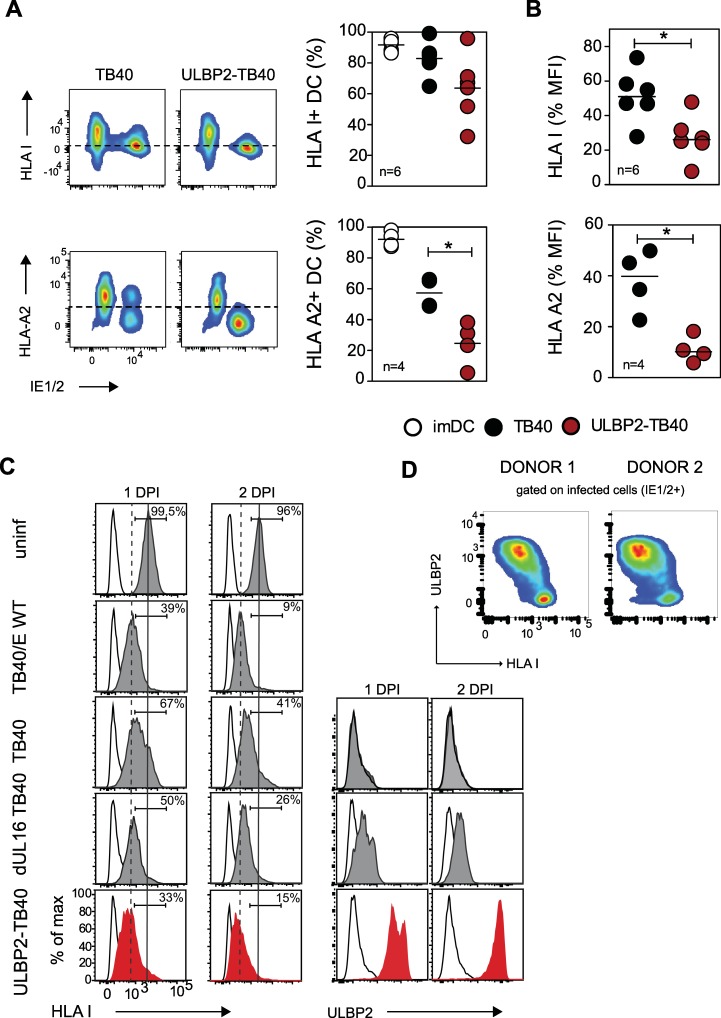
ULBP2-TB40 infected cells have lower MHC class I surface expression than TB40 infected cells. (A,B) Representative plots showing HLA class I expression using pan-HLA-A,B,C antibody (HLA I) or HLA-A02:01 antibody labeling of infected (IE1/2+) DC. Graphs provide (A) percentage and (B) median fluorescence intensity (MFI) of HLA class I positive uninfected immature DC (imDC) (white circles), or TB40 (black circles) and ULBP2-TB40 (red circles) infected DC of 4 to 6 donors. Horizontal bars represent medians. Data were analyzed with Mann-Whitney t-test. *, P < 0.05. (C) Representative staining for HLA class I on uninfected HFF or HFF infected with 1 PFU/cell of the indicated viruses at day 1 or 2 p.i. The histograms on the right depict ULBP2 expression on TB40, dUL16 TB40 (grey) or ULBP2-TB40 infected cells (red) at the indicated time points p.i. Black line, isotype controls. (D) HLA class I and ULBP2 staining gated on ULBP2-TB40 infected fibroblasts (IE1/2-positive cells) derived from two different donors.

Previous studies suggested that ULBP2 can co-stimulate CD8+ T cells via the NKG2D receptor [54]. In order to understand how ULBP2-TB40 infected DC activate CD8+ T cells, we first analyzed the maturation phenotype of the DC. Maturation of HCMV-infected DC in terms of CD80, CD86 and HLA DR expression was found to be impaired–as described by others [51–53]–to the same extent for both viruses (S9 Fig). Interestingly, HLA class I downregulation was more pronounced in ULBP2-TB40 infected DC (Fig 5A and 5B), which became particularly obvious when the surface expression level of HLA-A02:01 molecules was analyzed (Fig 5A and 5B, bottom panels). To attribute this phenotype to the individual genetic alterations introduced into the ULBP2-TB40 strain, we performed infection experiments with different viruses. In particular, we included the original TB40/E strain (referred to as TB40/E WT), harboring a complete complement of the MHC immunoevasins (US2, US3, US6, US11), and the strain dUL16 TB40 that lacks ORF UL16 as well as ORFs US1 to US6. When analyzed 1 and 2 days post infection (p.i.) the strongest HLA-I downregulation was observed for TB40/E WT infected fibroblast (Fig 5C), substantially more pronounced than in TB40-infected cells, reflecting the effect of the US2 to US6 genes on HLA-I surface expression. Remarkably, HLA-I downregulation in ULBP2-TB40 infected cells was as strong as in cells infected with the TB40E WT strain (Fig 5C, compare second panel and bottom panel), indicating an additional effect of ULBP2 expression (Fig 5C, bottom panel on the right). The dUL16 TB40 virus induced an intermediate phenotype (Fig 5C, second last panel), most likely due to upregulation of ULBP2 as a consequence of missing UL16 (Fig 5C, second last panel on the right). The degree of HLA-I downregulation in dUL16 TB40 and ULBP2-TB40 infected cells correlated with ULBP2 expression on the surface of these cells (Fig 5C). Intriguingly, plotting of HLA class I and ULBP2 surface expression for ULBP2-TB40 infected cells indicates that there is an inverse relationship of the expression of these molecules (Fig 5D). Taken together, these results show that expression of the NKG2D ligand ULBP2 increases MHC class I down-regulation in HCMV-infected cells.

### ULBP2 triggers loss of NKG2D from the surface of HCMV-specific T cells

**Fig 6:**
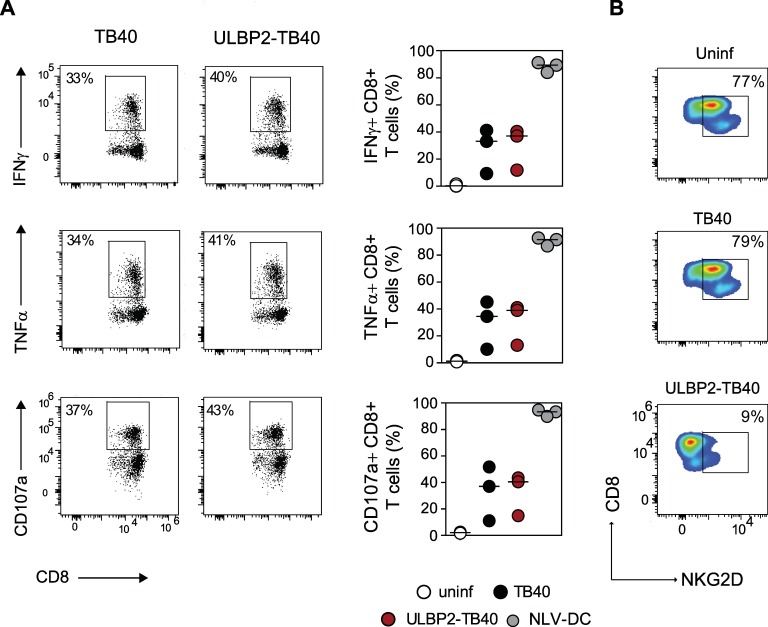
HCMV-specific CTL activated by ULBP2-expressing infected DC strongly downregulate NKG2D. (A) Representative staining of pp65-CTL for IFNγ, TNFα and CD107a after co-culturing with infected DC derived from one donor. Graphs represent compiled data for 3 donors as percentages of IFNγ, TNFα and CD107a positive pp65-CTL after co-culturing with uninfected DC (white circles), with DC infected with TB40 (black circles) or ULBP2-TB40 (red circles) or with pp65-peptide loaded DC (NLV-DC; grey circles). Bars represent medians. (B) Flow cytometry analysis of NKG2D expression on pp65-CTL after co-culturing with uninfected or TB40 or ULBP2-TB40 infected DC. Representative plots for one out of three donors analyzed. Data are representative of one of two independent experiment performed.

The observed lower surface expression of HLA-I molecules in ULBP2-TB40 infected cells was expected to impair antigen presentation, leading to less CD8+ T cell activation than upon stimulation with TB40-infected cells. However, this was in contrast to the results presented in Figs 3 and 4. To investigate this puzzle, we decided to examine in more detail the role of ULBP2 in T cell activation. To this end, we took advantage of a CD8+ T cell clone [63] specific for the peptide NLVPMVATV of the HCMV antigen pp65 (pp65-CTL) and co-cultured it with ULBP2-TB40 or TB40 infected DC. We noticed that a comparable percentage of the pp65-CTL became activated as assessed by IFNγ and TNFα intracellular cytokine staining, independent of which virus was used for the infection of DC (Fig 6A). In addition, comparable results were observed for the degranulation capacity (CD107a) of the pp65-CTL (Fig 6A). Data were confirmed with DC derived from three different donors. Next, we analyzed the expression of NKG2D on the surface of the pp65-CTL. Whereas the majority of the pp65-CTL expressed NKG2D when co-cultured with uninfected or TB40 infected DC, NKG2D surface expression was strongly diminished in the presence of ULBP2-TB40 infected DC (Fig 6B). The finding was confirmed with pp65-specific CD8+ T cells obtained by expansion with pp65-peptide loaded DC (S10A and S10B Fig). We conclude that although the outcome in terms of T cell activation is similar for DC infected with either strain, the underlying activating mechanism is different for T cells co-cultivated with ULBP2-TB40 infected DC and involves the NKG2D receptor as indicated by its strong, almost complete downregulation.

## Discussion

In this report we propose a novel vaccination strategy that can be applied to the development of an HCMV vaccine. The ULBP2 ligand for the NKG2D receptor that is present on both NK and CD8+ T cells was expressed by an HCMV strain with the intention to stimulate innate as well as adaptive immunity. We investigated the properties of the resulting recombinant HCMV strain by *in vitro* assays using *ex vivo*-derived primary human immune cells and provide a first *in vivo* assessment in a humanized mouse model.

An important goal in development of a live vaccine is attenuation. To this end, we endowed the HCMV strain with the ULBP2 gene, encoding a ligand for the activating NKG2D receptor of NK cells, and deleted the viral gene UL16, which normally counteracts the surface exposure of ULBP2 as well as of several other NKG2D ligands [38, 40]. This secured that there is no interference with ULBP2 expression and, even in the unlikely case that the ULBP2 gene is accidentally inactivated, the HCMV strain will remain susceptible to NK cell control. Although NK cells are well known for their contribution to contain acute HCMV infection, these innate immune cells cannot deploy their full efficacy because a series of NK cell evasion functions encoded by wild-type HCMV strains dampen their activity [64]. UL16 it is one of the important immunoevasins, which confers NK cell resistance of HCMV infected cells [39, 40]. By breaking NK cell evasion, cells infected with the ULBP2-expressing HCMV strain were expected to become highly vulnerable to recognition and elimination by NK cells. This was exactly what we observed when we performed cytotoxicity assays (Fig 1C and 1D). Consequently, spread of the ULBP2-expressing viral strain was strongly limited in the presence of NK cells (Fig 1E and 1F). These data suggest that the attenuation of the ULBP2-expressing HCMV strain will not depend on pre-existing immunity against HCMV, but on the presence of NK cells. Indeed, we detected human NK cells in the humanized mice which could contribute to the low viral load observed in animals receiving the ULBP2-expressing viral strain. This is supported by the fact that in a humanized mouse model for Epstein–Barr virus (EBV) human NK cells were capable to control the infection [65]. One difference between the EBV and HCMV infection models is that B cells in humanized mice support productive EBV infection, whereas important target cells of productive HCMV infection such as human hepatocytes, endothelial and epithelial cells are missing [66] and thus, primarily a latent HCMV infection is established at low level in few myeloid cells present in these animals [44]. Such limitations hamper the investigation of HCMV-specific immune responses in humanized NSG-A2 mice [67]. We found it nevertheless interesting that there was a trend towards a higher frequency of mature CD57+ NK cells in humanized mice receiving the ULBP2-TB40 strain when compared to the group receiving TB40. Emergence of a subset of CD57+ NK cells occurs in humans during primary HCMV infection [46]. Whether the observation for the CD57+ NK cells points to a stronger expansion in mice injected with the ULBP2-TB40 strain warrants further investigation. Similarly encouraging was the detection of CMV-specific IgM antibodies and a comparable frequency of B cells and CD4+ T cells as well as CMV-specific CD8+ T cells in mice receiving the ULBP2-TB40 and TB40 strains. Humoral immunity is one of the important factors in preventing congenital CMV infection and thus it is essential that a CMV vaccine can also provoke a strong antibody response. NSG mice transplanted with HSC are not ideal for analyzing humoral immunity. In several publications impaired antibody responses were reported [49], and only low levels of IgM were seen and no IgG [50] as in our study. Despite this constraint, our results indicate that ULBP2 expression by the ULBP2-TB40 strain does not interfere with the ability to elicit an IgM response. Induction of CMV-specific IgG antibodies must be analyzed in the future in novel humanized models that support antibody isotype switching [68–70]. We assess the observed HCMV-specific T and B cell responses as very promising results, particularly in view of the low load of ULBP2-TB40 genomes in the humanized mice. However, our study can only provide a first assessment of the vaccination approach with the ULBP2-TB40 strain, and further investigation of the protective capacity of the immune response has to await the establishment of improved humanized mouse models for HCMV infection. As recently pointed out by Crawford et al. (2015) [67], BLT (bone marrow/liver/thymus) humanized mice [71, 72] may allow to reproduce acute HCMV infection and analysis of the specific immune responses much better.

Although attenuation is a central objective concerning safety of a live vaccine, too much attenuation could impair immunogenicity. Rapid control of the vaccine strain may for instance limit the production of viral antigens–below a level necessary for priming of the adaptive T cell response. To circumvent this problem, we chose an HCMV strain that lacked several of the MHC immunoevasins. Such strains modulate antigen presentation to a lesser extent than HCMV isolates expressing the full complement of immunoevasins [73, 74]. In view of these considerations, we value the comparable activation and proliferation of HCMV-specific T cells observed upon co-cultivation with ULBP2-TB40 and TB40 infected DCs (Figs 3 and 4C) as a positive result. As a matter of fact, we expected a stronger CD8+ T cell response, because there was a second reason to express ULBP2 by the HCMV strain. It has been reported that NKG2D - besides being an activating receptor on NK cells—is also a co-stimulatory receptor expressed on CD8+ T cells [54, 75]. Indeed, when we co-cultured HCMV-specific T cells with ULBP2-TB40 infected DC, strong downregulation of NKG2D was observed (Figs 6B and S10B Fig). This did not occur when the T cells were co-cultured with uninfected or TB40-infected DC. We concluded therefore that reduced NKG2D surface expression reflects triggering of the receptor in the T cells. Regulation of NKG2D expression has been described as a physiologic mechanism [76] and therefore NKG2D downregulation in our assays should not be misinterpreted as a negative effect as previously suggested [77–79]. Along the same line, we observed that the HCMV-specific T cells that we obtained in the expansion assays expressed high levels of NKG2D (S11 Fig), suggesting that initial NKG2D downregulation is transient and rather indicates co-stimulation and activation of the T cells.

The seemingly neutral outcome of ULBP2 expression with respect to the frequency of activated CD8+ T cells (Figs 3 and 4) can best be explained by masking of NKG2D-mediated costimulation, an effect imposed by the lower MHC-I surface level that came as unexpected consequence of ULBP2 expression (Fig 5). It is long known that under conditions of low MHC-I expression and limiting amounts of antigenic peptides, extensive activation of T cells cannot occur [80]; in particular, when it comes to priming of naïve CD8+ T cells because they have a higher activation threshold than memory or effector CD8+ T cells. Our data imply that precisely in this setting when only low peptide concentrations are present, costimulation via NKG2D can secure the induction of specific T cells. This view is supported by the results of our previous mouse studies, which indicated that immunization with MCMV strains expressing the mouse NKG2D ligand RAE-1γ provided stronger protective immunity than infection with wild-type MCMV [15, 32]. It will be of interest to understand how NKG2D ligands influence MHC-I surface expression. Interestingly, a previous publication reported that expression of the murine NKG2D ligand RAE-1ε in RMA cells decreases MHC class I expression as well [81], suggesting that this is a more general phenomenon applying to different NKG2D ligands. Unraveling the exact underlying mechanism is beyond the scope of this work and must be the subject of further studies.

An excellent safety profile would increase the acceptance of a potential HCMV vaccine and will be an important goal, particularly when vaccination of immunocompromised patients is considered. Further attenuation can probably be achieved by deletion of additional genes for the numerous viral immunomodulatory functions, although it has to be examined that this does not go at the expense of reduced vaccination efficacy. Another option to increase safety is the application of replication- and spread-deficient vaccine strains. Studies in the mouse model revealed that such strains preserve the ability to stimulate CMV-specific T cell immunity [82, 83], and in a guinea pig model of congenital CMV infection partial protection of pups was observed following immunization with a non-infectious guinea pig CMV BAC DNA vaccine [84] or with a non-replication-competent virus [85]. We have recently pioneered the development of conditional-replicating CMV strains [86], and this technique has already successfully been used to generate a conditionally replicating HCMV vaccine candidate, which is currently in phase 1 testing (ClinicalTrials.gov number, NCT01986010). The combination of the superior immunization approach presented here in this study with the safety features of a replication-deficient strain could lead to an HCMV vaccine that may even be safely applicable to immunocompromised patients. Additional preclinical studies are therefore warranted to further analyze the protective efficacy as well as the safety of such vaccination strategies against HCMV, particularly when improved humanized mouse models become available, ultimately paving the way for clinical trials.

## Materials and Methods

### Ethics statement

Buffy coat samples were obtained from the Institute for Transfusion Medicine at Hannover Medical School from voluntary healthy blood donors with known HCMV serological status. All materials and data were analyzed anonymously. All studies were performed in accordance with guidelines on human cell research and the approval of the Hannover Medical School Ethics Review Board. All animal research protocols were approved by the authorized Ethics Committee for Biomedical Research of the Clinical Hospital Rijeka and of the University of Rijeka (Cl. 003-08/12-01/40; No. 2170-24-01-12-03) in accordance with the guidance of the European Parliament (Directive 2010/63/EU) and Croatian Federal Law about animal protection (Official Gazette, 135/2006 and 47/2011).

### Isolation and culture of cells

PBMC were purified from buffy coat samples obtained from healthy blood donors using standard Biocoll (Biochrom) density gradient centrifugation according to a published protocol (Miltenyi Biotec). Cells were cryopreserved in fetal bovine serum (FBS) with 10% DMSO. Before NK cell isolation or prior to setting-up co-cultures, PBMC were thawed and cultured overnight with 500 IU/ml interleukin-2 (IL-2) (ImmunoTools) in X-VIVO 15 medium (Lonza) supplemented with 10% FBS and 5% human serum (inactivated at 56°C; Sigma Aldrich). Individual cell subsets (CD56+ NK cells, CD8+ T cells and CD14+ monocytes) were isolated using human cell isolation kits (Miltenyi Biotec). NK cells were negatively selected, while CD8+ T cells and monocytes were positively selected. Purity of selection was analyzed by flow cytometry and was more than 94%. The pp65-specific T cell clone (CMV-CTL; kindly provided by L. Hambach) was thawed 2 days before setting up assays in MEM-alpha medium (Lonza) supplemented with 120 IU/ml of IL-2 and 10% human serum. The CMV-CTL was tested for the absence of mycoplasma and its specificity was determined by intracellular cytokine staining and proliferation assays using pp65-peptide loaded cells as positive control. Human monocyte-derived dendritic cells (DC) were generated using a standard protocol [87]. Briefly, isolated monocytes were cultured for 5 days in X-VIVO 15 medium supplemented with recombinant granulocyte macrophage colony-stimulating factor (GM-CSF) and interleukin-4 (IL-4) (50 ng/ml each; ImmunoTools). After 5 days, DC were used for infection or were matured for 24 h by supplementing the medium with maturation cocktail: recombinant human TNF-α (200 IU/ml), IL-1β (5 ng/ml), IL-6 (10 ng/ml), Prostaglandin E2 (1 μg/ml) (ImmunoTools). Two hours before establishing co-cultures with PBMC or CD8+ T cells, mature DC were loaded with a peptide pool spanning the entire CMV pp65 antigen (20 μg/ml; JPT Peptide Technologies). Primary human foreskin fibroblasts (HFF) were generated following standard methods as described [88] and were maintained in DMEM containing 10% FBS, penicillin (100 units/ml), streptomycin sulfate (100 μg/ml), and 2 mM L-glutamine.

### Viruses and infections

For the generation of the recombinant ULBP2-TB40 virus the ULBP2 ORF was amplified using primers 5´-GTCGGTACCGTCGCAGTCTTCGGTCTGACCACCGTAGAACGAGAGCTCCACCATGGCAGCAGCCGCCGCTACC-3´ and 5´-CCCGGATCCCTCTCCTCAGATGCCAGGGAGGATGAAG-3´ and an ULBP2 cDNA clone (Open Biosystems; Genbank accession number: BC034689), and was cloned (via KpnI and BamHI) between the MCMV major immediate-early promoter sequence (corresponding to nucleotides 182849 to 183094 of the MCMV Smith Strain (Genbank accession number NC004065.1)) and a kanamycin resistance (KanR) cassette flanked by FRT sites. The whole insert was amplified with primers 5´-GACACCGGGCTCCATGCTGACGTAGGTACCGACTGGGGTCAAAAGCCTTTAAACGGTACTTTCCCATAGC-3´ and 5´-CTTATAGCAGCGTGAACGTTGCACGTGGCCTTTGCGGTTATCCGTTCAGGAACACTTAACGGCTGA-3´, and inserted into the BAC-cloned genome of the HCMV strain TB40/E [36] by red-α, -β, -γ-mediated recombineering as described previously [89] replacing ORF UL16. The KanR cassette was excised by FLP recombinase. Correct insertion was verified by restriction analysis (BglII and NotI) and sequencing (with primers 5´-GGCGATGCGGTATCGCGCACA-3´ and 5´-GACACCTGTTCGTCCAGAATC-3´). The mutant lacking ORF UL16 that was derived from the BAC-cloned TB40/E strain was described previously [41]. All virus stocks were produced by propagation on HFF. Briefly, supernatant was harvested at day 6 p.i., and virus was pelleted by ultracentrifugation (100,000 × g, 1 h) and finally resuspended in X-VIVO 15 medium. Viral titers were determined using standard plaque assay on HFF. DC and fibroblasts were infected at an MOI of 3 and 1, respectively, followed by centrifugal enhancement (800 × g, 30 min).

### NK cell cytotoxicity assay

HFF were infected with TB40 or ULBP2-TB40 as described above. One day p.i. the infection rate of the HFF was determined by flow cytometry following intranuclear staining with an Alexa Fluor 488-conjugated CMV IE1/2 antibody (Merck Millipore). Cells were cryopreserved and thawed at the day of establishing the cytotoxicity assay. Prior establishing co-cultures, cells were labeled with carboxyfluorescein succinimidyl ester (CFSE; eBioscience) and co-cultured with freshly isolated human NK cells at effector to target ratios of 16:1, 8:1 and 4:1 in X-VIVO 15 medium supplemented with 500 IU/ml IL-2. After 4 h incubation at 37°C, specific lysis was determined by flow cytometry as measured by determination of dead cells using the Fixable Viability Kit (L/D) (BioLegend). To evaluate the spontaneous death of cells, samples containing target cells only were used. Cytolytic activity of NK cells expressed as percentage of specific lysis was determined by the following calculation: (% CFSE^+^ L/D^+^ cell specific lysis—% CFSE^+^ L/D^+^ cell spontaneous lysis) / (100 - % CFSE^+^ L/D^+^ cell spontaneous lysis) × 100.

### Focus expansion assay

Focus expansion assays were established as previously described [41]. Briefly, infected HFF (10 cells/well; 3 d p.i.) were co-cultured with uninfected HFF (2 × 10^4^ cells/well). NK cells were added at the ratio 1:0.25 (HFF:NK cells). After 3 days, the cultures were fixed with 80% acetone, and stained with the IE1/2 antibody. Infected cells per focus of infection and number of infectious foci were counted using fluorescence microscopy. Each sample was performed in quadruplicates.

### Antibody blocking

Blocking of NKG2D of human NK cells *in vitro* was performed using the NKG2D-specific antibody clone 1D11 and isotype control mouse IgG1 (BioLegend) (both at 10 μg/ml). In addition, human Fc Block (25 μg/ml; BD Biosciences) was added.

### T cell expansion and intracellular cytokine staining assays

For expansion of CMV-specific T cells, CD8+ T cells (10^5^ cells/well) isolated from HLA-A*02:01 HCMV-seropositive healthy donors were co-cultured with autologous monocyte-derived DC (uninfected, infected with respective viruses or mature peptide-loaded) and with γ-irradiated autologous CD14- feeder cells (2–3 × 10^5^ cells/well). Co-cultures were set up in 96-well plates at the ratio 1:10 (DC:T cell) in X-VIVO 15 medium supplemented with 5% human serum and IL-2 (500 IU/ml), IL-7 and IL-15 (10 ng/ml each; ImmunoTools). Every 2 days fresh medium containing cytokines was replenished. After 7 days, expanded cells were transferred to 48-well plates, and freshly prepared DC and feeder cells were added. After 2 weeks, the percentage of pp65-specific CD8+ T cells was determined by flow cytometry following staining with the PE-conjugated tetramer HLA-A*02.01-NLVPMVATV (MoBiTec). For intracellular cytokine staining, the expanded T cells, PBMC or the pp65-specific CTL clone were stimulated in 96-well V-bottom plates for 6 h with DC loaded with the pp65 peptide pool or the NLVPMVATV peptide or with uninfected/infected DC for PBMC and the pp65-specific T cell clone (at a ratio 1:10 [DC:T cells]). Protein transport inhibitor cocktail and a PE-Cy7-conjugated antibody against CD107a (clone H4A3; Biolegend) were added at the beginning of the assay. For surface staining we used PerCp-Cy5.5-conjugated anti-human CD3 (clone OKT3; eBioscience), PE-Texas Red-conjugated anti-human CD8 (clone 3B5; Thermo Fisher Scientific) and in case of expanded T cells additionally the PE-conjugated tetramer HLA-A*02:01-NLVPMVATV. After fixation with 3% paraformaldehyde for 5 min at RT and permeabilization with 0.1% saponin for 20 min at RT, FITC-conjugated (for expanded T cells) or PE-conjugated anti-human IFNγ (clone 4S.B3; eBioscience) and APC-conjugated anti-human TNFα Abs (clone Mab11; eBioscience) were used for staining. One million of splenocytes isolated from humanized mice were incubated with 1 μg of the peptide 316-VLEETSVML-324 (HLA-A2 restricted immunodominant epitope from the CMV IE1 antigen) for 5 h at 37°C, with brefeldin A (eBioscience) added for the last 4 h of stimulation. For evaluation of IFNγ production by NK cells, 5 × 10^6^ splenocytes were resuspended in 400 μl of RPMI medium supplemented with 10% FCS and IL2 (500 U/ml) and cultured for 5 h at 37°C in the presence of brefeldin A.

### Engraftment and infection of NSG-HLA-A2 mice

Humanization of mice was performed at the Helmholtz Center for Infection Research (Braunschweig, Germany). Briefly, fetal liver mononuclear cells were isolated over Ficoll-density gradients, and CD38- CD34+ cells were enriched using a MACS selection kit (Miltenyi Biotec). Newborn (3–5 days old) NOD.Cg-*Prkdc* ^*scid*^ *Il2rg* ^*tm1Wjl*^Tg(HLA-A2.1)1Enge/SzJ mice expressing human HLA-A*02:01 (NSG-A2) were irradiated with 0.7 Gy and 4–6 h after irradiation injected with 1 × 10^5^ CD34+ cells intrahepatically. 3 months post transfer engraftment of human cells was determined in blood. Humanization status ranged from 70–90% of human leukocytes (hCD45+ cells). Animals were randomly assigned to experimental groups with the humanization status being equal among the experimental groups. Mice were injected intraperitoneally with uninfected or TB40 or ULBP2-TB40 infected DC (3 × 10^5^ cells/mouse) generated from monocytes of HLA-A*02:01 positive HCMV seronegative donors. Mice were daily treated with subcutaneous injections of recombinant human granulocyte colony-stimulating factor (G-CSF) (3.6 μg/mouse s.c.; Filgrastim HEXAL). Mice were boosted after 7 days with freshly prepared, uninfected or infected DC (3 × 10^5^ cells/mouse).

### Detection of CMV-specific IgM by ELISA

Analysis of CMV-specific IgM antibodies in serum of infected animals was performed using anti-CMV IgM Human in vitro ELISA kit (Abcam) according to the manufacturer’s instructions. Briefly, a 96-well plate was precoated with CMV antigens to bind cognate antibodies. Control and test samples were added to the wells in duplicates. Serum was isolated from blood using density gradient centrifugation applying the standard PBMC isolation protocol (Miltenyi Biotec) and stored at -20°C. The following controls were included: CMV IgM positive control, CMV IgM negative control and CMV IgM cut-off calibrator. Following washing, a horseradish peroxidase (HRP) labelled anti-human IgM conjugate was added to the wells and after incubation as according to the instructions of the manufacturer, the reaction was stopped by adding an acidic stop solution. Absorbance at 450 nm was measured immediately using a TriStar Version 1.04 microplate reader (BertholdTech).

### Histology and immunohistochemistry

Sections from formalin fixed, paraffin-embedded spleens were stained with hematoxylin and eosin. Labelling of tissue sections was performed by an anti-human CD3 (F7.2.38) Ab (Dako) followed by biotinylated goat anti-mouse IgG (BD Pharmingen), streptavidin covalently coupled to horseradish peroxidase conjugate (Roche Applied Science) and DAB chromogene (Dako). Slides were examined with an Olympus BX51 microscope, images were acquired by an Olympus digital camera (DP71) and analyzed using Cell^B software.

### Quantitative PCR analysis of HCMV DNA

DNA was isolated from spleen, liver, bone-marrow, blood and salivary glands using the ReliaPrep Blood gDNA Miniprep Kit (Promega) according to the manufacturer’s protocol. Presence of HCMV DNA was determined by 7500 Fast Real-Time PCR system (Applied Biosystems) using a predefined two step PCR: 1 cycle at 95°C for 15 min followed by 40 cycles at 95°C for 15 s and 60°C for 1 min using Power SYBR Green PCR master mix and TaqMan Universal PCR mix (Applied Biosystems). To detect viral DNA primers specific for gB (gB_Fw 5’-AGG TCT TCA AGG AAC TCA GCA AGA and gB_Rv 5’-CGG CAA TCG GTT TGT TGT AAA) and a TaqMan gB probe with TAMRA quencher (6FAM—AAC CCG TCA GCC ATT CTC TCG GC—TAMRA) was used as previously described [90]. For the standardization curve we used the gB and IE1-TOPO TA pCR2.1 plasmid (kindly provided by S. Boppana; University of Alabama, Birmingham, AL).

### Flow cytometry

For flow cytometry analysis the following antibodies were used: anti-human Alexa Fluor 700-conjugated CD80 (L307.4; BD Biosciences), Brilliant Violet 510 conjugated CD86 (IT2.2; BioLegend), PE-conjugated CD209 (5H10; eBioscience), PE-Cy7-conjugated CD14 (61D3; eBioscience), PE-CF594-conjugated HLA-DR (G46-6; BD Biosciences), APC- -conjugated CD3 (OKT3; eBioscience), Alexa Fluor780-conjugated CD8 (SK1; conjugated HLA-ABC (W6/32; eBioscience), BV510-conjugated CD1a (HI149; BD Biosciences), APC-conjugated HLA-A2 (BB7.2; eBioscience), PE-conjugated ULBP2/5/6 (165903; R&D Systems), APC eBioscience), PE-Cy7–conjugated CD19 (HIB19; eBioscience), PE-conjugated CD56 (CMSSB; eBioscience), APC-conjugated PD-1 (MIH4; eBioscience), FITC-conjugated CD57 (TB01; eBiosceince), FITC-CD45RA (HI100; eBioscience), PE-Cy7-CCR7 (3D12; eBioscience), PE-Cy7-CD62L (DREG-56; eBioscience) and FITC-CD45RO (UCHL1; eBioscience). As isotype controls we used PE‑conjugated mouse IgG2a (R&D Systems) and APC-conjugated mouse IgG2b (eBioscience). For dead cells exclusion Zombie NIR Fixable Viability Kit (BioLegend) was used. Intranuclear staining with an Alexa Fluor 488-conjugated IE1/2-specific antibody (Merck Millipore) after PFA fixation and permeabilization with 75% ice-cold ethanol was used to detect infected cells. For evaluation of the phenotype of CD8+ T cell we used anti-human APC-conjugated NKG2D (1D11; eBioscience). CMV-specific CD8+ T cells from blood and spleen of humanized mice were analyzed on days 14 and 18 p.i, respectively, using the PE-conjugated pp65-specific tetramer HLA-A*02.01-NLVPMVATV and the APC-conjugated IE1-specific tetramer HLA-A*02:01- VLEETSVML synthesized by the NIH tetramer core facility. Flow cytometry was performed using FACSVerse, FACSAria or LSRII (BD Biosciences), and data were analyzed using FlowJo software (Tree Star).

### Statistical analyses

Statistical analyses were performed with GraphPad Prism5 software and statistical tests used are indicated in the figure legends. No statistical method was used to predetermine sample size, and data analysis was not blinded. Exact sample sizes and number of independent experiments performed are indicated in each figure legend. No samples were excluded. All error bars represent SEM or range as noted in the figure legends. Differences were considered to be statistically significant for P values <0.05.

## Supporting Information

S1 Fig Insertion of the ULBP2 gene does not affect replication of the ULBP2-TB40 virus in vitro. HFF were infected with 1 PFU per cells with TB40 (black) and ULBP2-TB40 (red) viruses. At indicated time points supernatants were harvested, and viral titers were determined by plaque assay. The values at day 0 represent the inocula.
(PDF)

S2 Fig NK cells decrease the number of infectious foci per well in cultures with ULBP2-TB40 infected cells. Focus expansion assays were set up using TB40 or ULBP2-TB40 infected HFF and primary NK cells of 6 donors as described in Fig 1E. A focus of infection was defined as a cluster of at least 3 and not more than 60 infected cells. Each donor was analyzed in quadruplicates. Results are mean numbers of infectious foci per well ±SEM.
(PDF)

S3 Fig Percentage and phenotype of NK cells in humanized mice. Humanized mice were treated as described in Fig 2. (A) Percentage of CD56+ NK cells out of the total CD3-negative cell population (CD3-CD19-) in livers for the respective groups. Each circle represents the result for one animal; horizontal bars indicate medians. (B, C) Percentage of CD57+ (B) and IFNγ+ NK cells (C) in spleens of animals from the indicated groups. Differences between the groups were analyzed by Mann-Whitney t-test. *, P < 0.05; not significant (ns), P > 0.05.
(PDF)

S4 Fig Infected DC led to the priming of HCMV-specific CD8+ T cells in humanized mice. Immunization was performed with TB40 or ULBP2-TB40-infected DC in humanized mice (as described in Fig 2). Dot plots indicate staining of IE1-tetramer+ lymphocytes (IE1-tet) isolated from blood of 3 animals from each group 2 weeks after immunization. Percentages of IE1 (upper graph) and pp65-specific CD8+ T cells (lower graph) for animals of the groups receiving DC infected with the respective viruses. Differences between the groups were not significant as analyzed by Mann-Whitney t-test. ns, P > 0.05.
(PDF)

S5 Fig Functionality and phenotype of antigen-specific CD8+ T cells. Humanized mice were injected with infected DCs as described in Fig 2. (A) On day 18 p.i. the frequency of IE1-specific CD8+ T cells was analyzed by IE1-tetramer staining of splenocytes derived from animals of the respective groups. Representative staining for cells of one animal analyzed from each group. Graph at right provides the compiled data for IE1-tetramer+ CD8+ T cells in spleen of animals for the indicated groups. (B) Intracellular cytokine staining to evaluate percentage of IFNγ-positive CD8+ T cells after 6 h stimulation with the IE1 peptide. Graph shows cumulative data for IFNγ-positive CD8+ T cells from the indicated groups. (C) Percentage of naive (CD62L+CD45RO-) and terminally differentially effector memory (TEMRA; CD62L-CD45RO-) CD8+ T cell subsets in spleen from animals in the indicated. Data were analyzed with Mann-Whitney t-test. ns, P > 0.05.
(PDF)

S6 Fig Expansion of pp65-specific CD8+ T cells using peptide loaded mature DC as positive control. Representative dot plots depicting percentages of pp65-specific CD8+ T cells from one donor before expansion and after 14 days of co-culture with uninfected autologous DC or pp65-peptide loaded mature DC (pp65-mDC). Lower plots, staining with irrelevant tetramer as negative control (irrel-tet). The graph at right is compiled data from 4 donors. Horizontal bars are medians. Data are representative for one of two independent experiment performed.
(PDF)

S7 Fig Phenotype of expanded T cells. Representative dot plots of an experiment performed with cells from one donor indicating percentage of naive (N; CCR7+CD45RA+), central memory (CM; CCR7+CD45RA-), effector memory (EM; CCR7-CD45RA-) and terminally differentiated effector memory (TEMRA; CCR7-CD45RA+) CD8+ T cells before expansion and after 14 days of co-culture with TB40 or ULBP2-TB40 infected DC. Graphs display compiled data for four donors as percentages of (A) CM or EM CD8+ T cells and (B) CM or EM pp65-specific CD8+ T cells expanded with TB40 (black circles), ULBP2-TB40 (red circles) infected DC or pp65-peptide loaded DC (grey circles). (C, D) Percentage of PD-1+ CD8+ T cells (C) and PD1+ pp65-specific CD8+ T cells (D). Data obtained with cells from individual donors are connected by lines. Data are representative of one of two independent experiment performed. Statistical analysis was done with one-way ANOVA Friedman test followed by Dunn’s Multiple Comparison test. *, P < 0.05; ns, P > 0.05.
(PDF)

S8 Fig CD4 T cell responses in PBMC stimulation assay. (A) Percentage of IE1/2-positive DC 1 day after infection with 3 PFU per cell of TB40 (black circles) and ULBP2-TB40 (red circles). Compiled data for DC of four donors are given, horizontal bars represent medians. (B, C) Intracellular cytokine staining for IFNγ (B) and TNFα (C) expression of CD4 T cells (CD3+CD8-) upon co-cultivation of PBMC with autologous monocyte-derived DC that remained uninfected or were infected as indicated. Graphs are compiled data of experiments performed with cells of 4 HCMV-seropositive donors. Data generated with cells of individual donors are connected by lines and are representative of one of three independent experiment performed. Statistical analysis was done with one-way ANOVA Friedman test followed by Dunn’s Multiple Comparison test. *, P < 0.05; ns, P > 0.05.
(PDF)

S9 Fig Maturation phenotype of infected DC. (A) Representative staining for CD80, CD86 and HLA-DR expression on TB40 or ULBP2-TB40 infected DC (IE1/2-positive cells) and on uninfected DC matured as described in Materials and Methods. Graphs at right provide compiled data for DC of 6 donors. Results obtained with DC of one donor are connected by lines. (B) Median fluorescence intensity (MFI) of CD80, CD86 and HLA-DR for TB40 (black circles) and ULBP2-TB40 (red circles) infected DC relative to MFI values of uninfected DC in the same cultures (IE1/2-negative bystander cells) (n = 6 donors). Horizontal bars represent medians. Representative experiment of two of seven independent experiments performed. Data were analyzed using one-way ANOVA Friedman test followed by Dunn’s Multiple Comparison test. *P < 0.05; ns, P > 0.05.
(PDF)

S10 Fig Stimulation of pp65-specific T cells by ULBP2-expressing HCMV-infected DC leads to NKG2D downregulation. pp65-specific CD8+ T cells were obtained by co-culturing CD8+ T cells from HLA-A2+, HCMV seropositive donors with pp65-peptide loaded autologous DC as described in Materials and Methods. Representative plots of pp65-specific CD8+ T cells (derived from one donor) after co-culturing with autologous uninfected DC or DC infected with the indicated viruses show (A) IFNγ, TNFα and CD107a intracellular staining and (B) NKG2D surface expression. Similar data were obtained using cells derived from three different donors.
(PDF)

S11 Fig CD8+ T cells expanded with HCMV infected DC express NKG2D. (A) Histograms depict NKG2D expression on CD8+ T cells from one donor before expansion and after 2 weeks of co-culture with uninfected, TB40 or ULBP2-TB40 infected DC or pp65-peptides loaded mature DC (pp65-mDC). Black lines indicate staining with isotype antibody. (B) Compiled data depicting percentages of NKG2D positive CD8+ T cells from 4 donors before expansion and after expansion with the different DC. Data obtained with cells from one donor are connected by lines.
(PDF)
