# Supplementary figures and images for: Activation of Innate and Adaptive Immunity by a Recombinant Human Cytomegalovirus Strain Expressing an NKG2D Ligand
Source: PLoS Pathog. 2016 Dec 1;12(12):e1006015. doi: 10.1371/journal.ppat.1006015 (PMC5131914; doi:10.1371/journal.ppat.1006015)

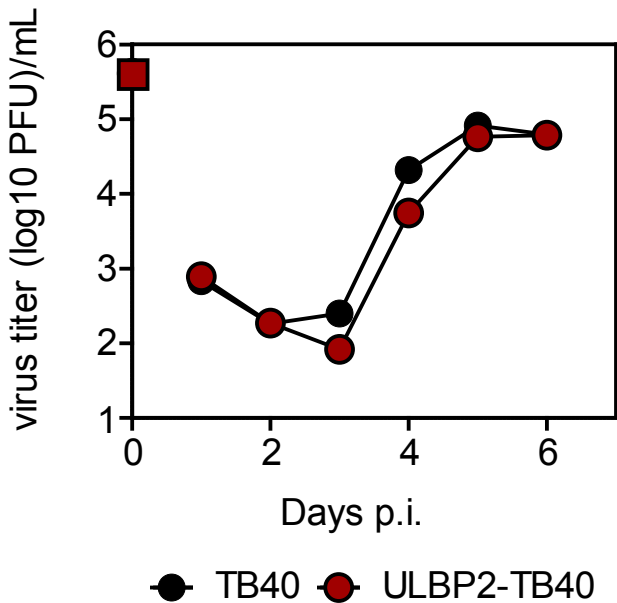

Supplement: S1 Fig — HFF were infected with 1 PFU per cells with TB40 (black) and ULBP2-TB40 (red) viruses. At indicated time points supernatants were harvested, and viral titers were determined by plaque assay. The values at day 0 represent the inocula. (PDF) [file ppat.1006015.s001.pdf]

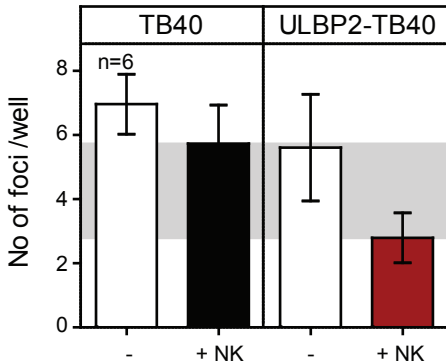

Supplement: S2 Fig — Focus expansion assays were set up using TB40 or ULBP2-TB40 infected HFF and primary NK cells of 6 donors as described in Fig 1E. A focus of infection was defined as a cluster of at least 3 and not more than 60 infected cells. Each donor was analyzed in quadruplicates. Results are mean numbers of infectious foci per well ±SEM. (PDF) [file ppat.1006015.s002.pdf]

**A**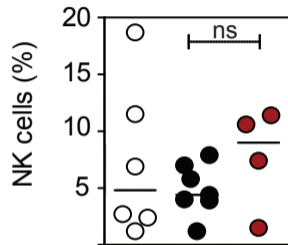**B**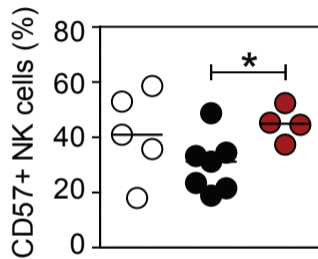**C**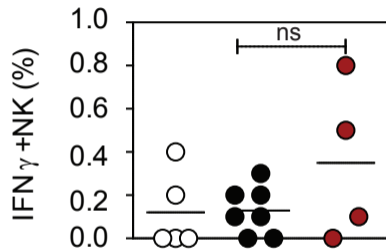

○ uninf    ● TB40    ● ULBP2-TB40

Supplement: S3 Fig — Humanized mice were treated as described in Fig 2. (A) Percentage of CD56+ NK cells out of the total CD3-negative cell population (CD3-CD19-) in livers for the respective groups. Each circle represents the result for one animal; horizontal bars indicate medians. (B, C) Percentage of CD57+ (B) and IFNγ+ NK cells (C) in spleens of animals from the indicated groups. Differences between the groups were analyzed by Mann-Whitney t-test. *, P < 0.05; not significant (ns), P > 0.05. (PDF) [file ppat.1006015.s003.pdf]

ULBP2-TB40

TB40

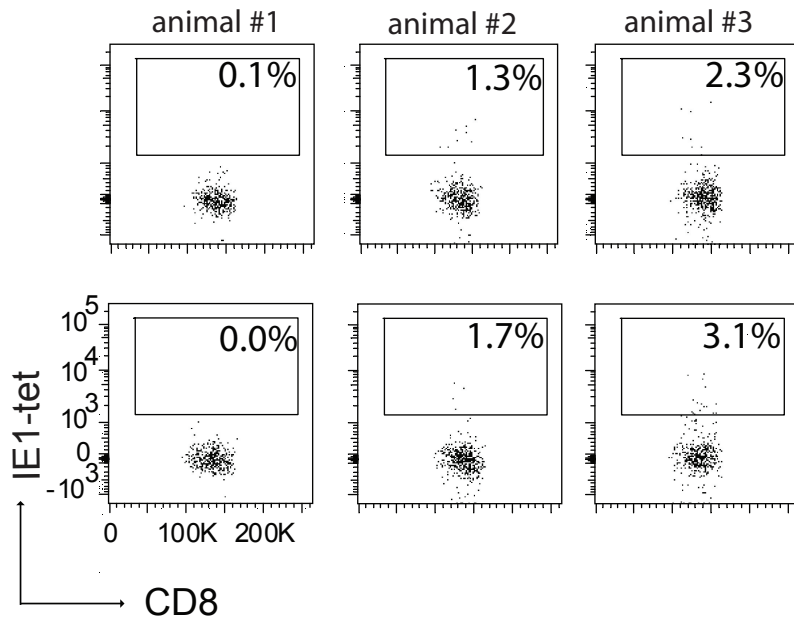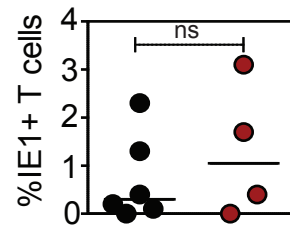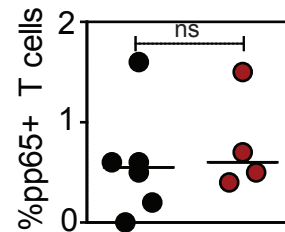

● TB40

● ULBP2-TB40

Supplement: S4 Fig — Immunization was performed with TB40 or ULBP2-TB40-infected DC in humanized mice (as described in Fig 2). Dot plots indicate staining of IE1-tetramer+ lymphocytes (IE1-tet) isolated from blood of 3 animals from each group 2 weeks after immunization. Percentages of IE1 (upper graph) and pp65-specific CD8+ T cells (lower graph) for animals of the groups receiving DC infected with the respective viruses. Differences between the groups were not significant as analyzed by Mann-Whitney t-test. ns, P > 0.05. (PDF) [file ppat.1006015.s004.pdf]

# A

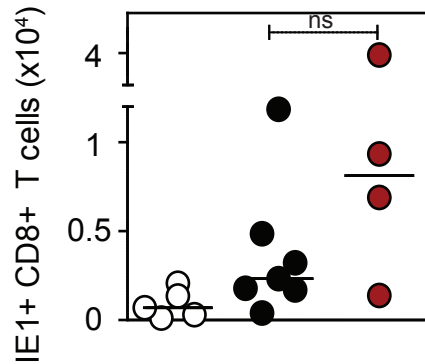

B

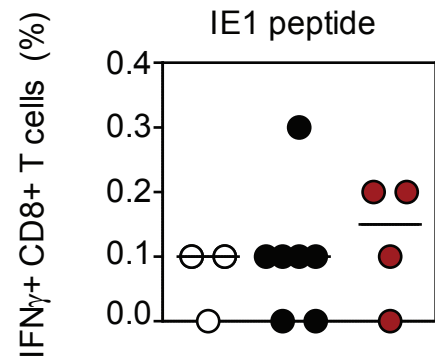

○ uninf    ● TB40    ● UI BP2-TB40

C

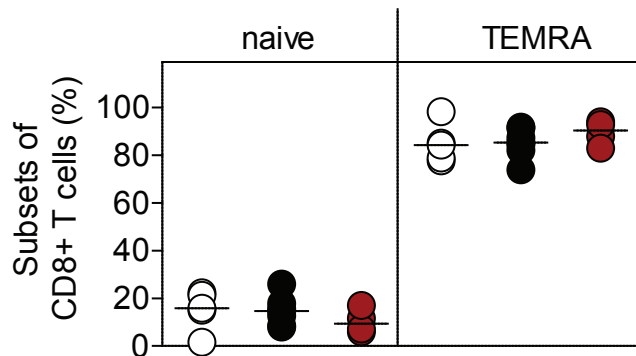

Supplement: S5 Fig — Humanized mice were injected with infected DCs as described in Fig 2. (A) On day 18 p.i. the frequency of IE1-specific CD8+ T cells was analyzed by IE1-tetramer staining of splenocytes derived from animals of the respective groups. Representative staining for cells of one animal analyzed from each group. Graph at right provides the compiled data for IE1-tetramer+ CD8+ T cells in spleen of animals for the indicated groups. (B) Intracellular cytokine staining to evaluate percentage of IFNγ-positive CD8+ T cells after 6 h stimulation with the IE1 peptide. Graph shows cumulative data for IFNγ-positive CD8+ T cells from the indicated groups. (C) Percentage of naive (CD62L+CD45RO-) and terminally differentially effector memory (TEMRA; CD62L-CD45RO-) CD8+ T cell subsets in spleen from animals in the indicated. Data were analyzed with Mann-Whitney t-test. ns, P > 0.05. (PDF) [file ppat.1006015.s005.pdf]

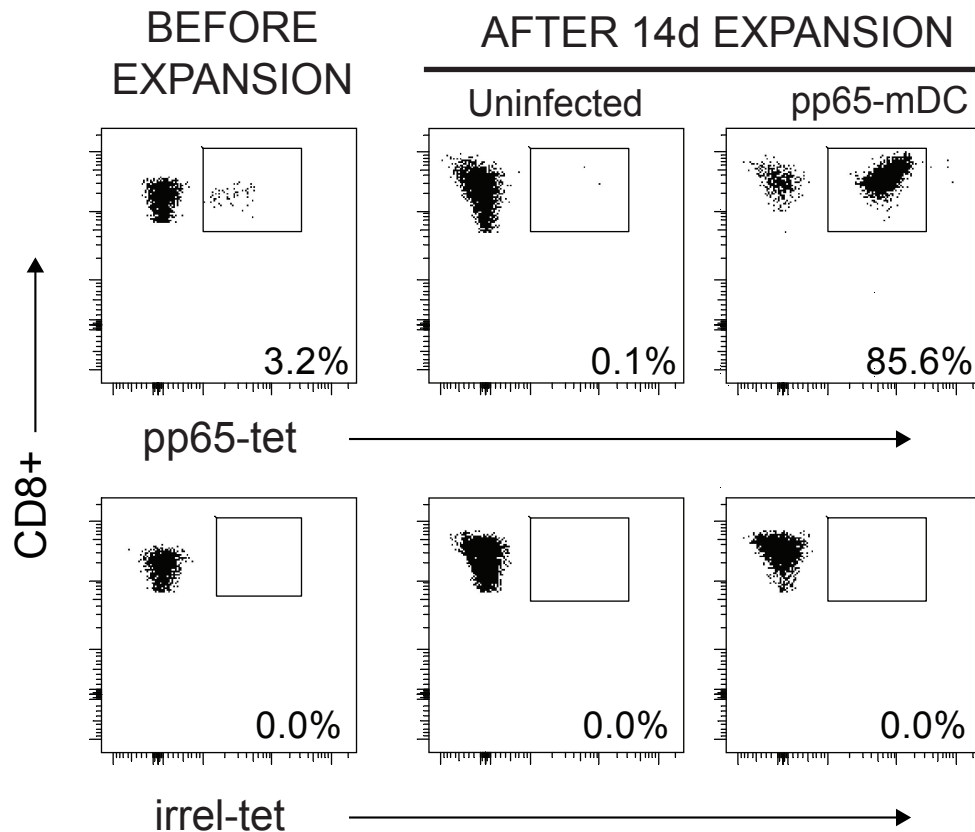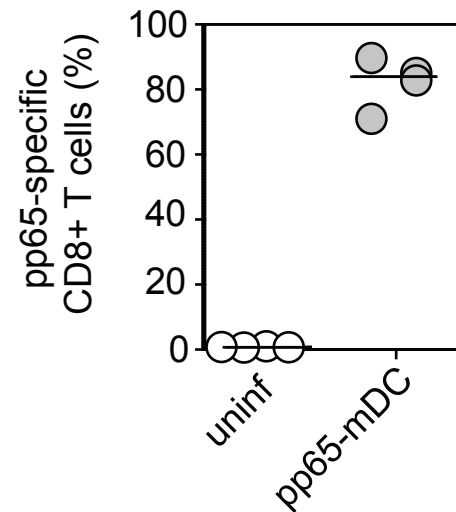

Supplement: S6 Fig — Representative dot plots depicting percentages of pp65-specific CD8+ T cells from one donor before expansion and after 14 days of co-culture with uninfected autologous DC or pp65-peptide loaded mature DC (pp65-mDC). Lower plots, staining with irrelevant tetramer as negative control (irrel-tet). The graph at right is compiled data from 4 donors. Horizontal bars are medians. Data are representative for one of two independent experiment performed. (PDF) [file ppat.1006015.s006.pdf]

**A**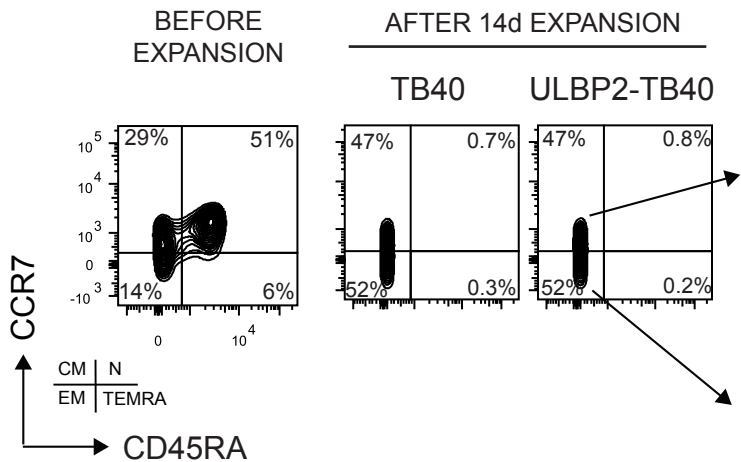**CENTRAL MEMORY**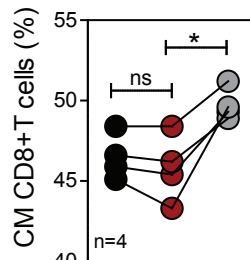**EFFECTOR MEMORY**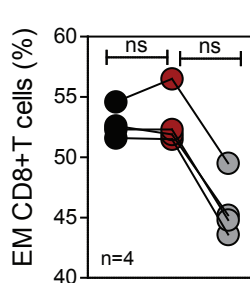**B**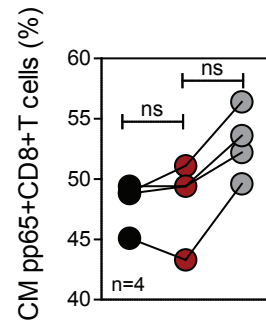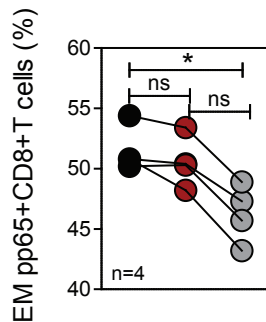**C**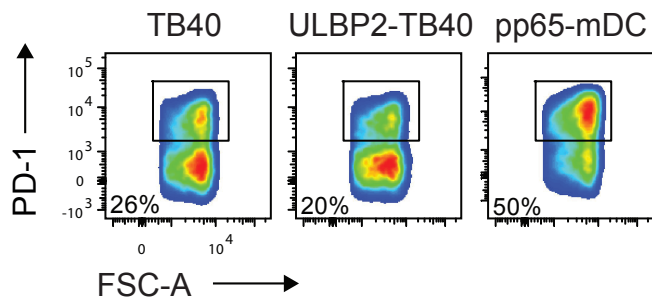**D**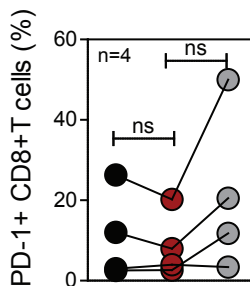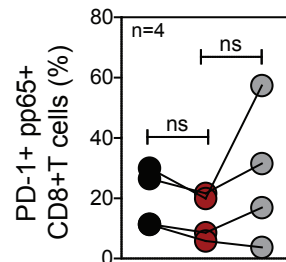

● TB40 ● ULBP2-TB40 ● pp65-mDC

Supplement: S7 Fig — Representative dot plots of an experiment performed with cells from one donor indicating percentage of naive (N; CCR7+CD45RA+), central memory (CM; CCR7+CD45RA-), effector memory (EM; CCR7-CD45RA-) and terminally differentiated effector memory (TEMRA; CCR7-CD45RA+) CD8+ T cells before expansion and after 14 days of co-culture with TB40 or ULBP2-TB40 infected DC. Graphs display compiled data for four donors as percentages of (A) CM or EM CD8+ T cells and (B) CM or EM pp65-specific CD8+ T cells expanded with TB40 (black circles), ULBP2-TB40 (red circles) infected DC or pp65-peptide loaded DC (grey circles). (C, D) Percentage of PD-1+ CD8+ T cells (C) and PD1+ pp65-specific CD8+ T cells (D). Data obtained with cells from individual donors are connected by lines. Data are representative of one of two independent experiment performed. Statistical analysis was done with one-way ANOVA Friedman test followed by Dunn’s Multiple Comparison test. *, P < 0.05; ns, P > 0.05. (PDF) [file ppat.1006015.s007.pdf]

**A**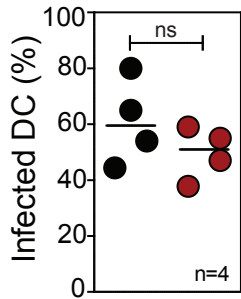**B**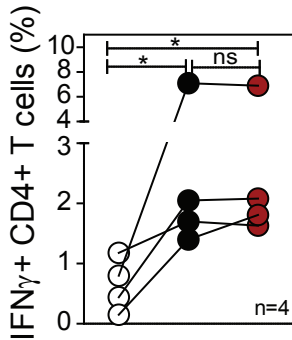**C**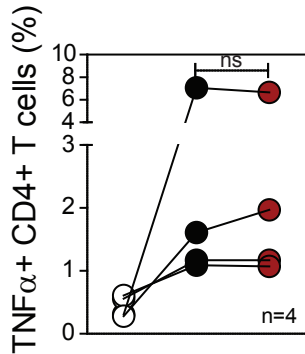

○ uninf    ● TB40    ● ULBP2-TB40

Supplement: S8 Fig — (A) Percentage of IE1/2-positive DC 1 day after infection with 3 PFU per cell of TB40 (black circles) and ULBP2-TB40 (red circles). Compiled data for DC of four donors are given, horizontal bars represent medians. (B, C) Intracellular cytokine staining for IFNγ (B) and TNFα (C) expression of CD4 T cells (CD3+CD8-) upon co-cultivation of PBMC with autologous monocyte-derived DC that remained uninfected or were infected as indicated. Graphs are compiled data of experiments performed with cells of 4 HCMV-seropositive donors. Data generated with cells of individual donors are connected by lines and are representative of one of three independent experiment performed. Statistical analysis was done with one-way ANOVA Friedman test followed by Dunn’s Multiple Comparison test. *, P < 0.05; ns, P > 0.05. (PDF) [file ppat.1006015.s008.pdf]

**A**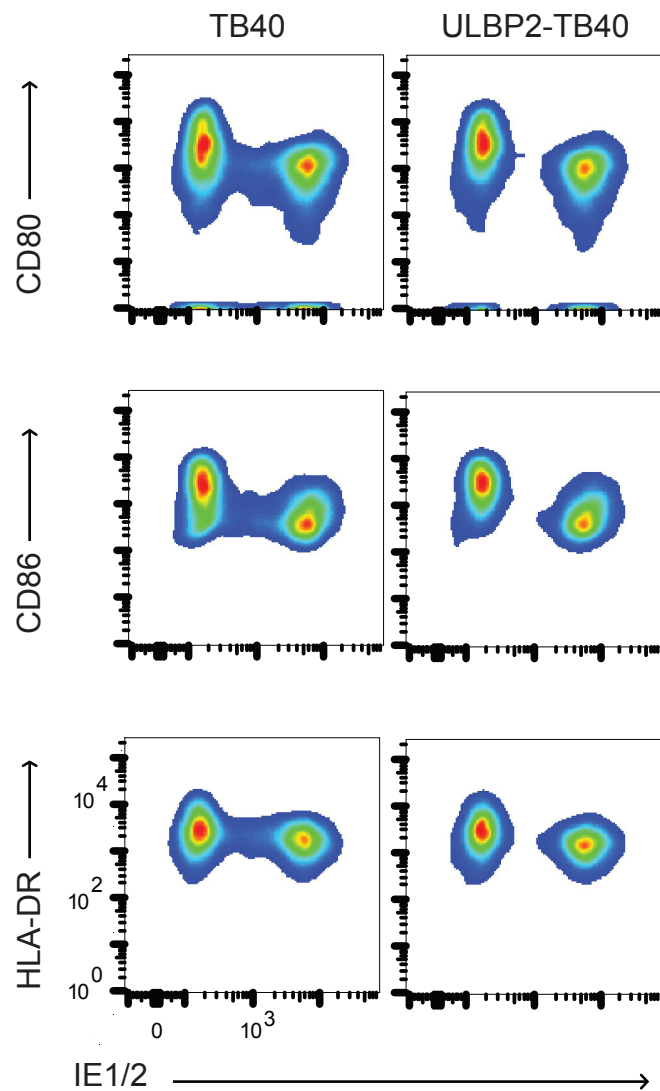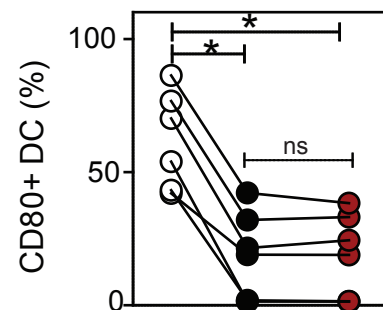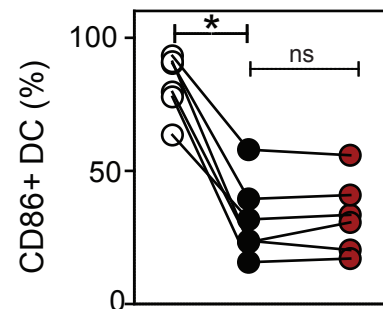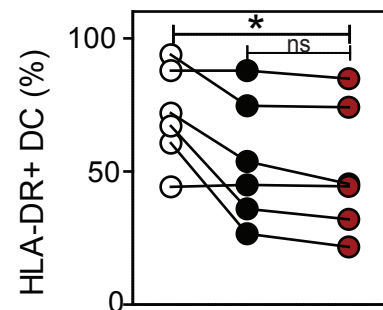**B**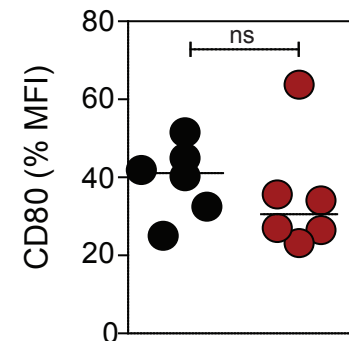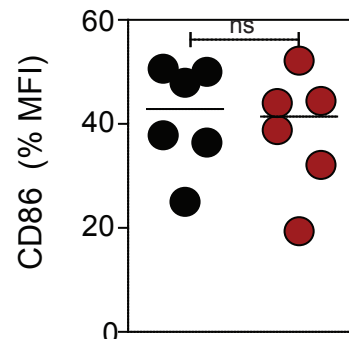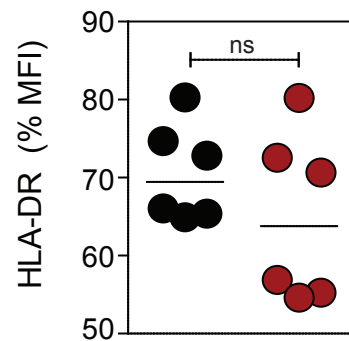

○ mature DC ● TB40 ● ULBP2-TB40

Supplement: S9 Fig — (A) Representative staining for CD80, CD86 and HLA-DR expression on TB40 or ULBP2-TB40 infected DC (IE1/2-positive cells) and on uninfected DC matured as described in Materials and Methods. Graphs at right provide compiled data for DC of 6 donors. Results obtained with DC of one donor are connected by lines. (B) Median fluorescence intensity (MFI) of CD80, CD86 and HLA-DR for TB40 (black circles) and ULBP2-TB40 (red circles) infected DC relative to MFI values of uninfected DC in the same cultures (IE1/2-negative bystander cells) (n = 6 donors). Horizontal bars represent medians. Representative experiment of two of seven independent experiments performed. Data were analyzed using one-way ANOVA Friedman test followed by Dunn’s Multiple Comparison test. *P < 0.05; ns, P > 0.05. (PDF) [file ppat.1006015.s009.pdf]

**A**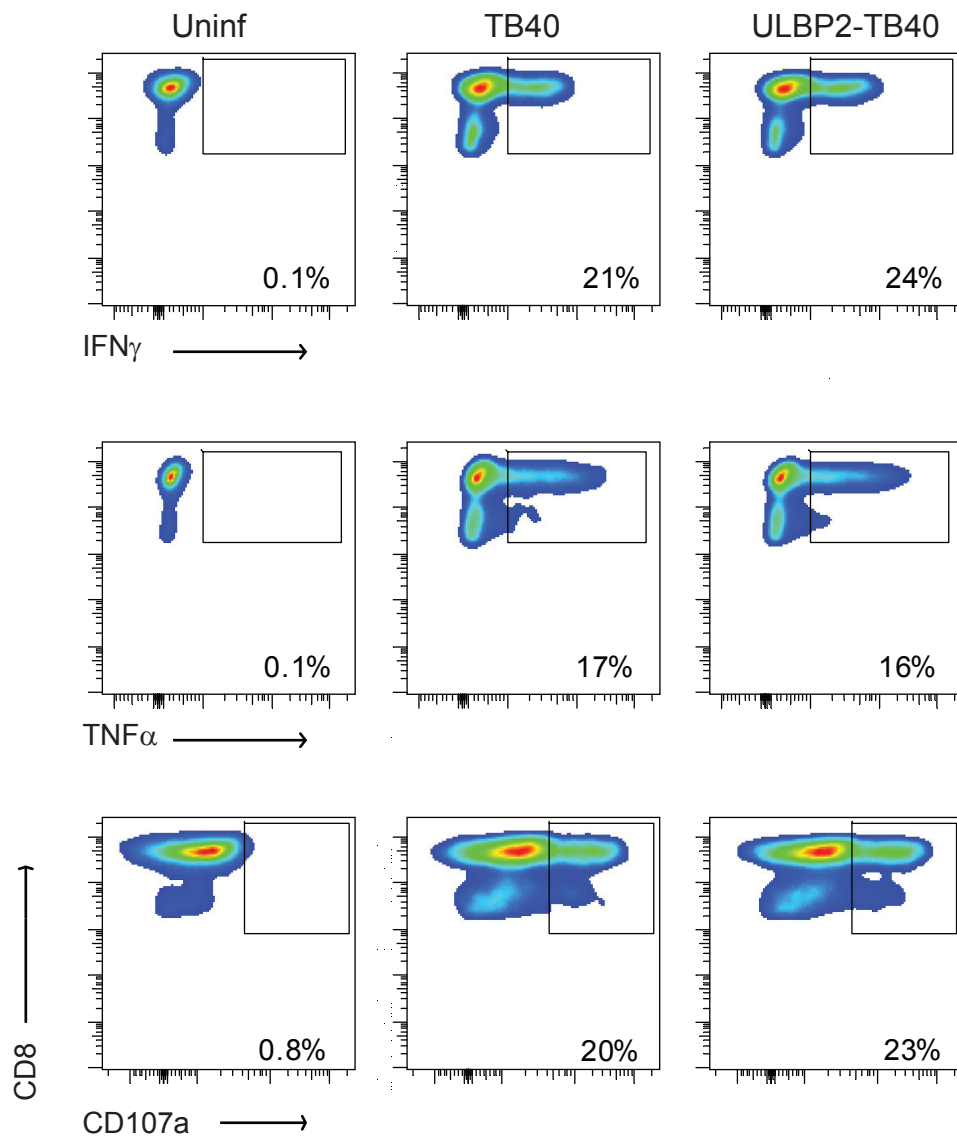**B**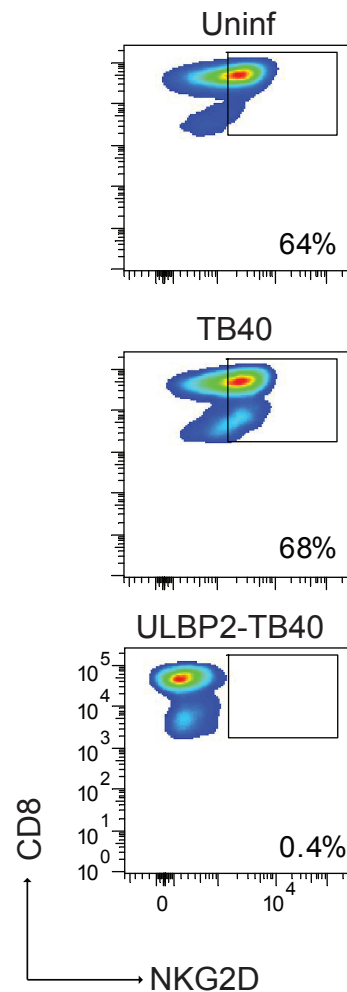

Supplement: S10 Fig — pp65-specific CD8+ T cells were obtained by co-culturing CD8+ T cells from HLA-A2+, HCMV seropositive donors with pp65-peptide loaded autologous DC as described in Materials and Methods. Representative plots of pp65-specific CD8+ T cells (derived from one donor) after co-culturing with autologous uninfected DC or DC infected with the indicated viruses show (A) IFNγ, TNFα and CD107a intracellular staining and (B) NKG2D surface expression. Similar data were obtained using cells derived from three different donors. (PDF) [file ppat.1006015.s010.pdf]

**A**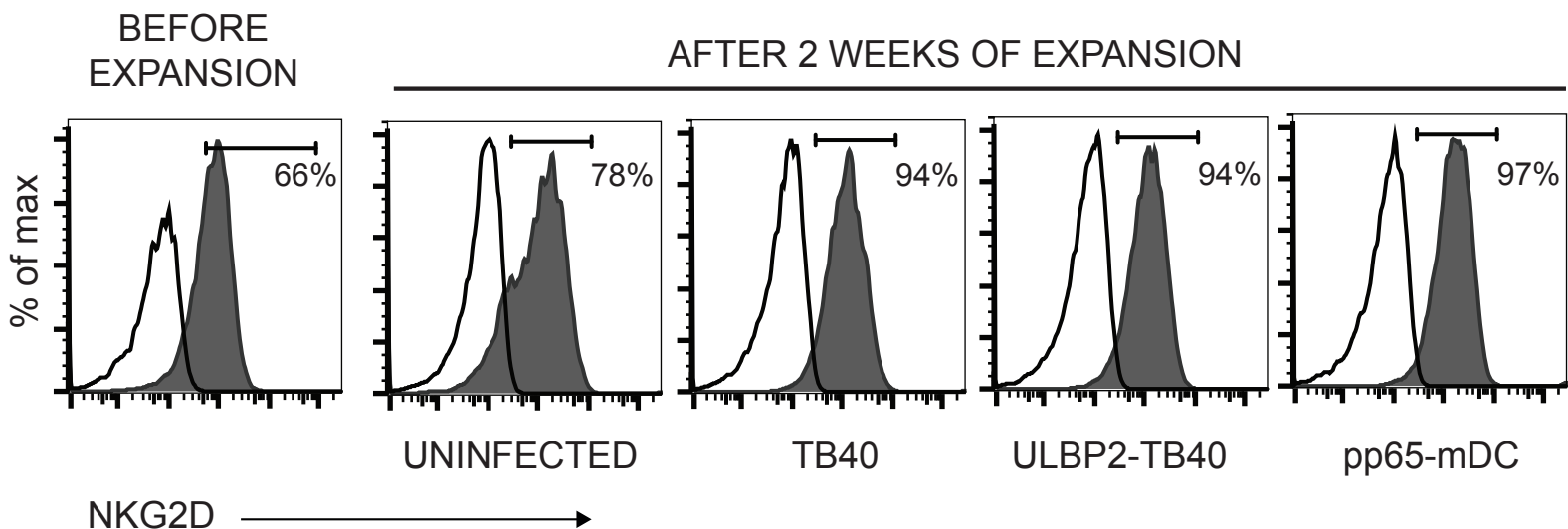**B**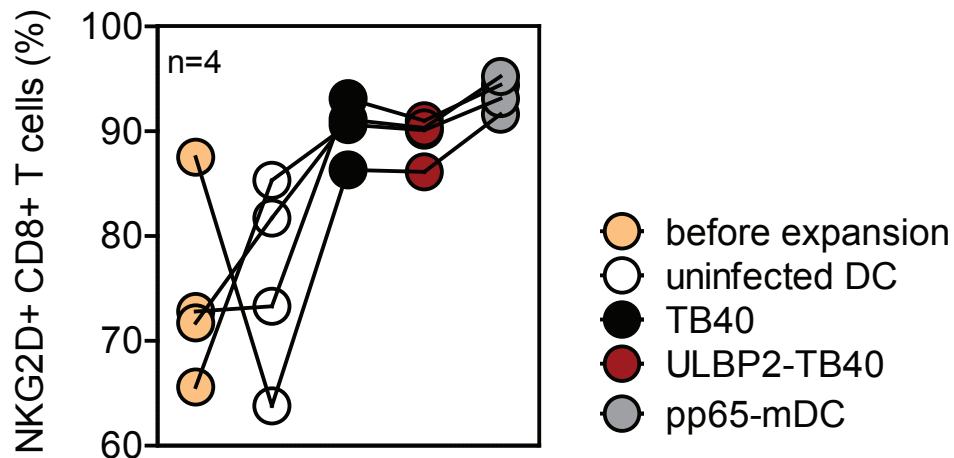

Supplement: S11 Fig — (A) Histograms depict NKG2D expression on CD8+ T cells from one donor before expansion and after 2 weeks of co-culture with uninfected, TB40 or ULBP2-TB40 infected DC or pp65-peptides loaded mature DC (pp65-mDC). Black lines indicate staining with isotype antibody. (B) Compiled data depicting percentages of NKG2D positive CD8+ T cells from 4 donors before expansion and after expansion with the different DC. Data obtained with cells from one donor are connected by lines. (PDF) [file ppat.1006015.s011.pdf]
